# Succinate and inosine coordinate innate immune response to bacterial infection

**DOI:** 10.1371/journal.ppat.1010796

**Published:** 2022-08-26

**Authors:** Ming Jiang, Zhuang-gui Chen, Hui Li, Tian-tuo Zhang, Man-jun Yang, Xuan-xian Peng, Bo Peng

**Affiliations:** 1 Center for Proteomics and Metabolomics, State Key Laboratory of Bio-Control, Guangdong Key Laboratory of Pharmaceutical Functional Genes, School of Life Sciences, Southern Marine Science and Engineering Guangdong Laboratory (Zhuhai), Sun Yat-sen University, Higher Education Mega Center, Guangzhou, People’s Republic of China; 2 Laboratory for Marine Biology and Biotechnology, Qingdao National Laboratory for Marine Science and Technology, Qingdao, People’s Republic of China; 3 Institute of Animal Science, Guangdong Academy of Agricultural Sciences, Guangzhou, People’s Republic of China; University of North Carolina at Chapel Hil, UNITED STATES

## Abstract

Macrophages restrict bacterial infection partly by stimulating phagocytosis and partly by stimulating release of cytokines and complement components. Here, we treat macrophages with LPS and a bacterial pathogen, and demonstrate that expression of cytokine IL-1β and bacterial phagocytosis increase to a transient peak 8 to 12 h post-treatment, while expression of complement component 3 (C3) continues to rise for 24 h post-treatment. Metabolomic analysis suggests a correlation between the cellular concentrations of succinate and IL-1β and of inosine and C3. This may involve a regulatory feedback mechanism, whereby succinate stimulates and inosine inhibits HIF-1α through their competitive interactions with prolyl hydroxylase. Furthermore, increased level of inosine in LPS-stimulated macrophages is linked to accumulation of adenosine monophosphate and that exogenous inosine improves the survival of bacterial pathogen-infected mice and tilapia. The implications of these data suggests potential therapeutic tools to prevent, manage or treat bacterial infections.

## Author summary

Bacterial infection is still one of the primary causes of death, especially in the context of exhausted antibiotic reservoir to the growing cases of antibiotic resistance. Novel approach to manage bacterial infection is required. Here, we report a metabolism-based approach to manage immune response to fight against bacterial infection. We find that innate immune response to bacterial infection is constituted with two phases, namely innate cellular immunity and innate humoral immunity. The two phases are interchangeably regulated by two metabolites, inosine and succinate. Succinate promotes the production of inflammatory cytokines IL-1β while inosine downregulates IL-1β expression but increases complement component 3 expression in a time-dependent manner. This is a novel phenomenon that this two-phases innate immune response is required to clear bacterial infection, and is confirmed by the *in vivo* challenge of mice and fish with pathogenic bacteria in combination of succinate or inosine. We also report a novel mechanism that succinate and inosine competitively regulate HIF-1α activation. Thus, our study presents a novel feedback mechanism of immune response, and inosine could be a potential metabolite in tuning immune response towards bacterial infection.

## Introduction

In response to invasion by bacterial pathogens, infected organisms rely on the innate immune system to mount an inflammatory response, restrict bacterial growth and prevent uncontrolled infection [1]. When the inflammatory response fails to stop bacterial growth, inflammation can lead to chronic, persistent bacterial infection. On the other hand, a persistent high level of inflammation is linked to inflammation-associated chronic disease [2]. While inflammation plays a critical role in the innate immune response to pathogenic bacteria, it must be tightly regulated, to ensure optimal outcomes.

The innate immune system includes cellular and humoral components, which are primarily mediated by macrophage- and complement-dependent processes, respectively, both of which play crucial roles in fighting bacterial infection [3]. Macrophages promote phagocytosis of invading bacterial pathogens, as well as secretion of pro-inflammatory cytokines such as IL-1β, TNF-α, and IL-6, which promote cellular innate immunity [4–6]; macrophages also promote secretion of complement components, which regulate and promote humoral innate immunity [7]. Activated complement can also promote bacterial lysis or recruit inflammatory cells via anaphylatoxins such as C3a and C5a. Both cellular and humoral innate immune responses restrict bacterial infections in their early stages [3,8,9]. Therefore, macrophages may help coordinate and/or regulate the kinetics of the cellular and humoral components of the innate immune response to environmental cues [10]. While it has been proposed that IL-1β is a master regulator of early inflammatory and immune responses to infection [11], high and/or persistent increases in IL-1β have been associated with immune dysfunction and disease [12]. Complement system, where C3 is the central component, not only boosts innate immune response but also is critical for the function of T- and B- immune response [3,13]. Patients with genetic deficiency of complement system are suffering from recurrent bacterial infection [13]. The mechanisms that coordinate and regulate the cellular and humoral innate immune responses remain poorly understood and warrant further study.

Metabolomic analysis is a powerful tool for identifying phenotypic, diagnostic and therapeutic biomarkers [14,15]. We recently linked distinct host and bacterial phenotypes with specific metabolomes, which we refer to as “infective / anti-infective metabolomes” in host cells and “antibiotic-resistant/antibiotic-sensitive metabolomes” in bacteria. Furthermore, evidence was presented that exposure to specific exogenous metabolites can re-program “infective” to “anti-infective” or “antibiotic-resistant” to “antibiotic-sensitive” phenotypes, with corresponding metabolomic re-programming [16–19]

The phenotypes, functions, and activities of macrophages have been characterized extensively [4,9,20,21]. For example, in response to LPS, macrophages upregulate glycolysis and downregulate oxidative phosphorylation and tricarboxylic acid (TCA) cycle metabolism [9,22,23], which contribute to the attenuated secretion of inflammatory cytokines and reduced bacterial killing [15,24]. Succinate, a TCA cycle metabolite, inhibits prolyl hydroxylase, a negative regulator of hypoxia-induced factor 1α (HIF-1α), leading to higher levels of IL-1β [24]. Itaconate, another TCA cycle metabolite, limits expression of pro-inflammatory cytokines including IL-1β, through interaction with NRF2, glutathione, and IκBζ [25–28]. Serine metabolism supports IL-1β production in macrophages, while dimethyl fumarate targets glyceraldehyde-3-phosphate dehydrogenase (GAPDH) and aerobic glycolysis to downregulate IL-1β production [29–31]. These findings document a complex and as yet poorly understood metabolite-related regulation of both cellular and humoral components of the innate immune response.

Here, we investigated the kinetics of the macrophage response to bacterial pathogens and LPS *in vitro* and *in vivo*, focusing on IL-1β and C3 as markers of cellular and humoral innate immune responses, respectively, and associated metabolic signatures. Succinate and inosine were identified as critical metabolites, correlated with IL-1β and C3, respectively, as well as distinct phases of the innate immune response, each progressing with distinct response kinetics. Evidence is also presented that succinate and inosine differentially up- or down-regulate expression of IL-1β, through a feedback loop mediated by HIF-1α and its negative regulator prolyl hydroxylase. The findings provide insight into mechanisms that regulate and maintain balance between cellular and humoral components of the innate immune response to bacterial infection.

## Results

### Kinetics of phagocytosis and expression of IL-1β and C3 in LPS-stimulated macrophages

To explore the regulation of the cellular and humoral components of the innate immune response, RAW264.7-asc cells were exposed to LPS (100 ng/mL), and the kinetics of the pro-inflammatory response was monitored for up to 24 h post-treatment. Cellular parameters were monitored, including frequency of phagocytosis, expression of IL-1β and C3. LPS is a prototypical bacterial pathogen-associated molecule that triggers a time-limited pro-inflammatory response in macrophages. The experiments were performed in RAW264.7-asc cells, which express *ASC* protein ectopically to resemble bone marrow-derived macrophage (BMDM) cells [32,33].

**Fig 1:**
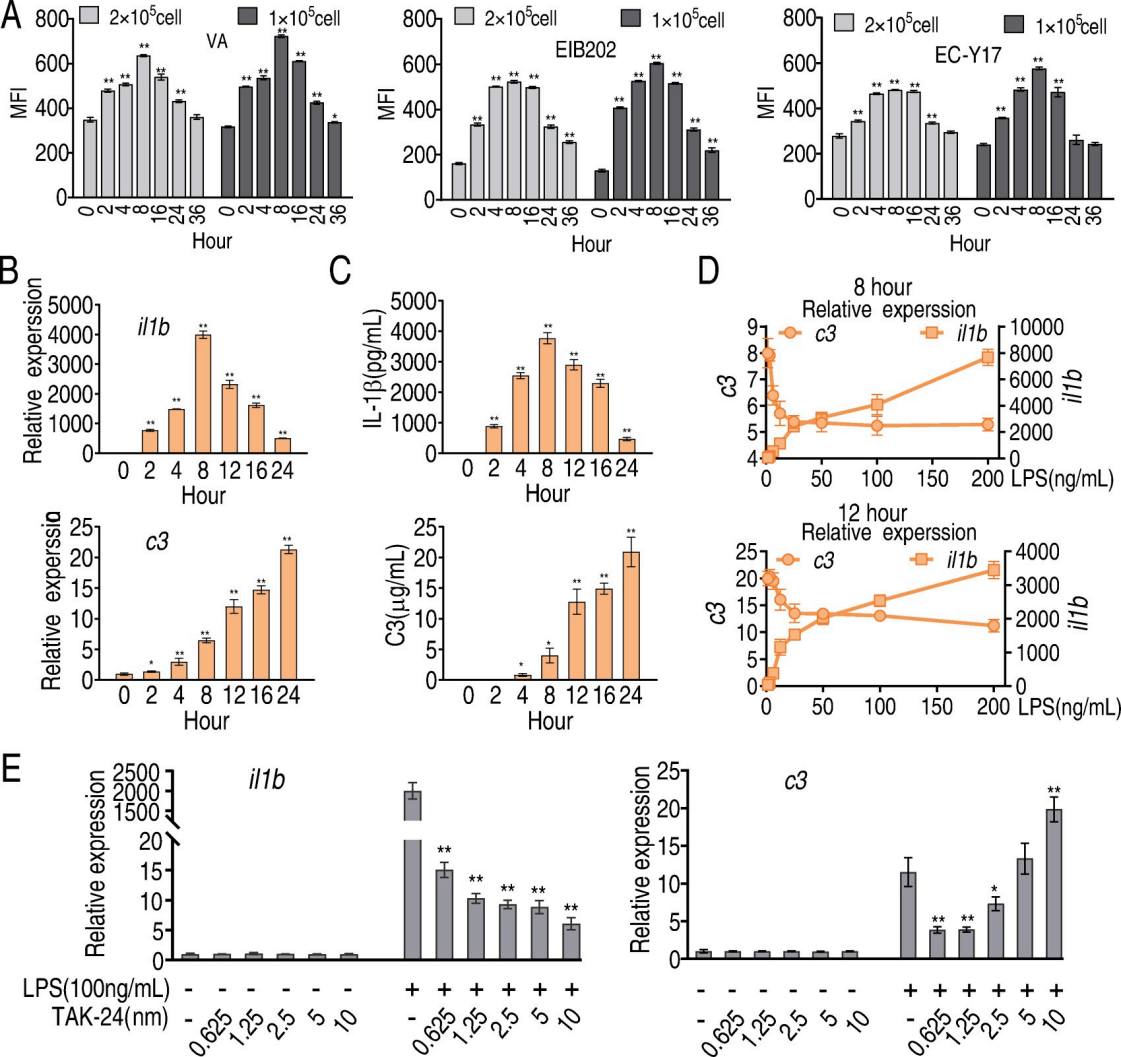
Phagocytosis, IL-1β and C3 in LPS-stimulated macrophages. A, Phagocytosis of FITC-labeled *V*. *alginolyticus* (VA), *E*. *tarda* (EIB202), or *E*. *coli* (Y17) by LPS-treated RAW264.7-asc cells. Cells were analyzed by flow cytometry and phagocytosis was estimated from mean fluorescence intensity at the indicated time points after LPS-stimulation. Three biological replicates were performed at each time point. B, Transcription of *il1b* and *c3* was estimated by qRT-PCR at the indicated time points after LPS stimulation. C, IL-1β and C3 were quantified in extracellular media by ELISA at the indicated time points after addition of LPS. D, Transcripts of *il1b* and *c3* were quantified by qRT-PCR in cells exposed for 8 or 12 h to the indicated concentration of LPS. E, Transcripts of *il1b* and *c3* were quantified in cells treated with 100 μg LPS. TLR4 Inhibitor TAK-24 at the indicated concentration, or LPS plus TLR4 inhibitor. Results (B-E) are displayed as mean ± SEM, and significant differences are identified (*p < 0.05, **p < 0.01) as determined by non-parametric Kruskal-Wallis one-way analysis with Dunn multiple comparison post hoc test.

Being treated with LPS for different time (2, 4, 8, 12, 16, 24, or 36 h), cells were then co-incubated with FITC-labeled *Vibrio alginolyticus* VS12G, *Escherichia coli* Y17, or *Edwardsiella tarda* EIB202 and phagocytosis was quantified by flow cytometry. The phagocytosis was increased along with duration of LPS treatment and peaked at and declined from 8 h post-treatment (Fig 1A). Similar pattern was detected for transcription of *il1b* as well as abundance of secreted IL-1β (Fig 1B and 1C). However, transcription of *c3* gradually increased up to 24 h after treatment, as did the abundance of secreted C3 (Fig 1B and 1C). These data suggest that the cellular and humoral components of the innate immune response occur with distinct kinetics, which may provide an optimized immune response to clear bacterial pathogens.

In cells exposed to an increasing concentration of LPS for 8 or 12 h, we observed positively-correlated increasing transcription of *il1b* and negatively-correlated decreasing transcription of *c3* (Fig 1D). Furthermore, increasing concentrations of an inhibitor TAK-24 of TLR4, the receptor for LPS, decreased transcription of *il1b* but increased transcription of *c3*, at a constant concentration of LPS (Fig 1E). These results suggest possible regulatory roles for *il1b* and *c3* during the innate immune response to LPS/bacterial pathogens. The following experiments explore whether and how the innate immune response is modulated by cellular metabolic state.

### The metabolome of LPS-treated RAW264.7-asc cells

**Fig 2:**
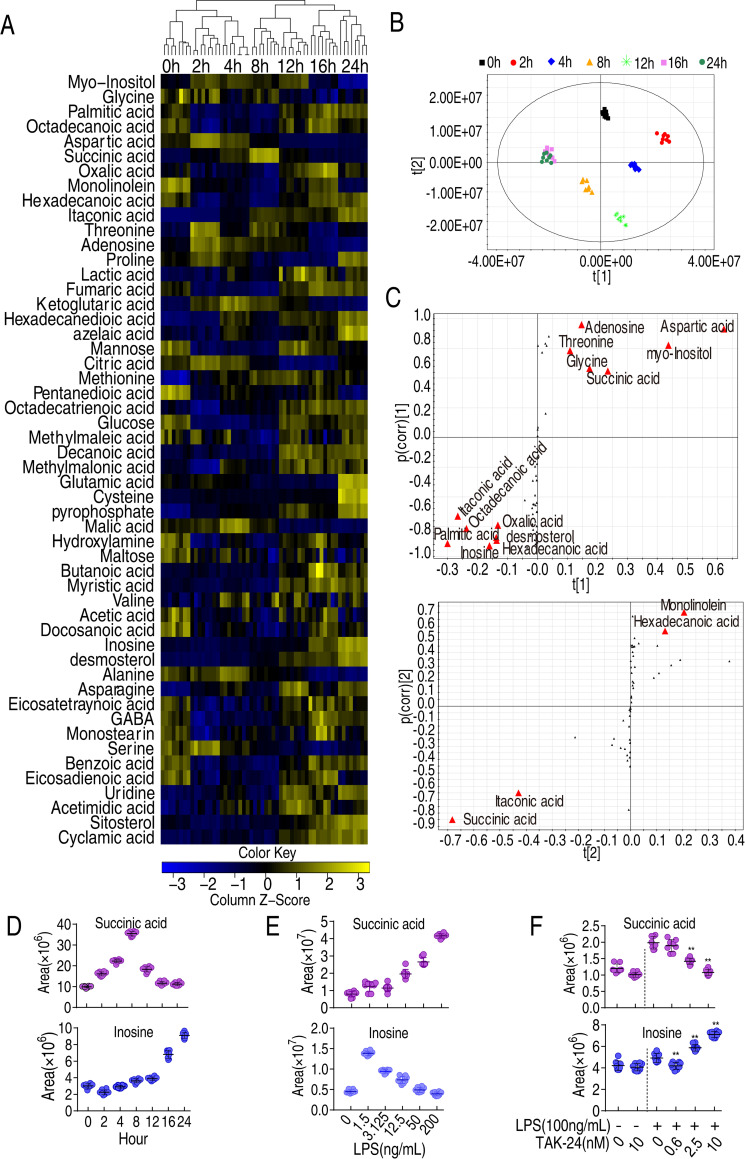
Dynamic changes in metabolomes of LPS-induced phagocytes. A, Heatmap showing abundance of metabolites in macrophages treated with LPS for the indicated length of time. B, PCA analysis of data shown in (A). Data from each time point are represented as a cluster, coded by color for each time point (see legend at top of panel B. C, S-plot generated from OPLS-DA, displaying metabolites of interest D, Relative abundance of succinate and inosine at the indicated time points after exposure to LPS. E, Relative abundance of succinate and inosine in RAW264.7-asc cells exposed to LPS at the indicated concentration for 8 h. F, Relative abundance of succinate and inosine of RAW264.7-asc cells in the presence of TLR4 inhibitor TAK-24.

We speculated that LPS triggers a metabolic shift associated with the innate immune response to bacterial pathogens. To gain insight into this response, a comprehensive metabolic analysis was performed from 0 to 24 h after exposure of RAW264.7-asc cells to LPS. Four biological and two technical replicates were performed at each time point, generating 56 data sets. Ribitol was used as an internal standard. A total of 53 metabolites whose abundance varied over the experimental time course were selected for further study. The correlation coefficients of technical replicates were between 0.996 and 0.998 (S1A Fig), indicating high reproducibility of the data. These metabolites were classified into five categories (S1B Fig). Differentially-abundant metabolites were displayed as a heat-map in (Fig 2A) and their Z-values were listed in (S2 Fig). Eleven metabolic pathway were enriched, where alanine, aspartate and glutamate metabolism, aminoacyl-tRNA biosynthesis, butanoate metabolism, and TCA cycle were the first four most significant pathways (S3 Fig), where alanine, aspartate and glutamate metabolism fules the TCA cycle, making the adjusted central metabolism is the core adjusted point.

To better identify metabolites that differentiate the groups, orthogonal partial least square discriminant analysis (OPLS-DA) was applied for multivariate analysis. The seven groups (*e*.*g*., time points) were clearly separated from each other. Principle component analysis identified t [1], which discriminated metabolites at earlier (0, 2, 4, and 8 h) and later (12, 16, and 24 h) time points, and t [2], which separated metabolites at 0, 12, and 16 h from at 2, 4, 8, and 24 h (Fig 2B). The cut-off values were set in OPLS-DA loadings plot for metabolites as ≥ 0.05 and 0.5 for absolute value of covariance p and correlation p(corr). Fifteen metabolites were identified as potential biomarkers of the innate immune response (Fig 2C). Furthermore, abundance of succinate peaked at approximately 8 h post-LPS treatment, as observed for IL-1β, while abundance of inosine increased gradually for up to 24 h post-treatment, as observed for complement factor C3 (Fig 2D and the other are listed in S4 Fig). The change in the abundance of succinate and inosine along with the change of IL-1β and C3 was confirmed in primary BMDM, which showed similar pattern (S5 Fig). We also observed that the abundance of inosine decreased and the abundance of succinate increased, as RAW264.7-asc cells were exposed to increased doses of LPS, from 1.5 ng/mL to 200 ng/mL (Fig 2E). However, treatment of TLR4 with the inhibitor TAK-24 decreased succinate abundance in a dose dependent manner while increased inosine in a does-dependent manner (Fig 2F). Based on these results, we explored the possibility that succinate and inosine play roles regulating and/or modulating the cellular and humoral components of the innate immune response, respectively, in LPS-stimulated macrophages.

### Comparison of succinate, inosine, IL-1β, and C3 abundance in LPS-stimulated macrophages

**Fig 3:**
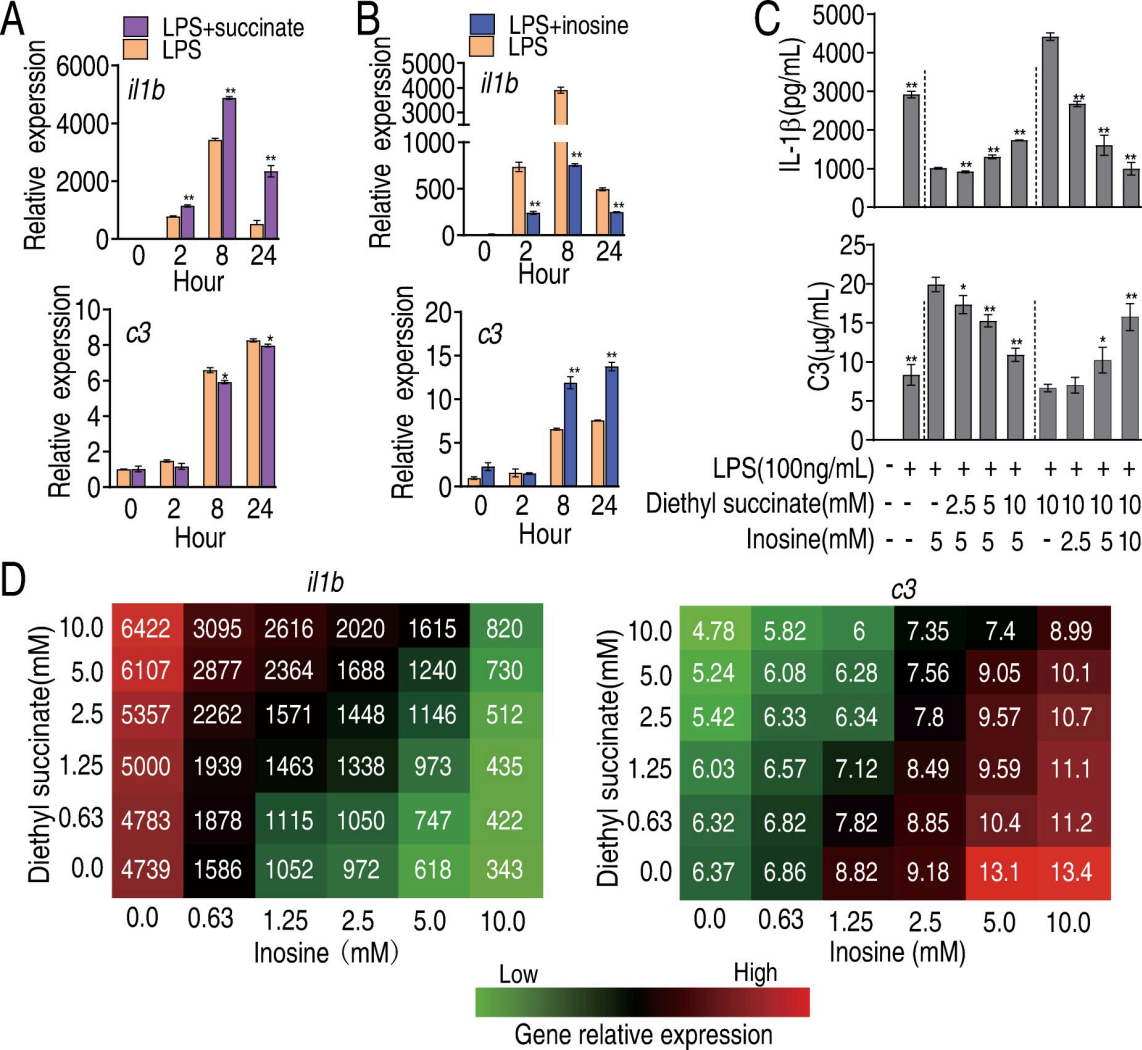
Comparative regulation to LPS-induced gene expression between succinate and inosine. A, Transcripts of *il1b* and *c3* were quantified in cells treated with LPS in the presence or absence of succinate as indicated. B, Transcripts of *il1b* and *c3* were quantified in cells treated with LPS in the presence or absence of inosine as indicated. C, Abundance of secreted IL-1β and C3 in cells treated with LPS, dimethyl succinate, inosine or LPS plus dimethyl succinate or LPS plus inosine or LPS plus inosine and dimethyl succinate at the indicated concentrations. D, Isobolograms showing antagonistic effects of inosine and succinate on expression of *il1b* and *c3*. Results (A-C) are displayed as mean ± SEM, and significant differences are identified (*P<0.05, **P<0.01) as determined by non-parametric Mann-Whitney U test (A and B) and non-parametric Kruskal-Wallis one-way analysis with Dunn multiple comparison post hoc test (C).

When RAW264.7-asc cells were exposed to exogenous dimethyl succinate, we observed higher transcription of *il1b*, and no similar effect on transcription of *c3* (Fig 3A). In contrast, in the presence of exogenous inosine, expression of *il1b* was lower while expression of *c3* was higher (Fig 3B). Simialr data was obtained in primary macrophages BMDM (S6 Fig). These results suggest that succinate and inosine have antagonizing effects on expression of *il1b* and *c3*.

Furthermore, in the presence of inosine (5 mM), which inhibits expression of IL-1β in LPS-treated macrophages, increasing concentration of succinate (2.5 mM, 5 mM, and 10 mM) partially counteracted the inhibiting effect of inosine. In contrast succinate (10 mM) stimulated expression of IL-1β, and increasing concentrations of inosine gradually restored the basal level of expression of IL-1β, in a dose-dependent manner (Fig 3C). Conversely, inosine-induced expression of C3 was inhibited in a dose-dependent manner by co-exposure to succinate, while increasing concentrations of inosine counteracted the inhibiting effect of succinate on C3 (Fig 3C). These relationships are summarized and quantified in isobolograms and the highest level of antagonism between the two metabolites was revealed (Fig 3D). We conclude that succinate and inosine play opposite (antagonistic) roles in regulating expression of *il1b* and *c3* in LPS-treated macrophages.

### Interactions between HIF-1α prolyl hydroxylase, HIF-1α, succinate, and inosine

**Fig 4:**
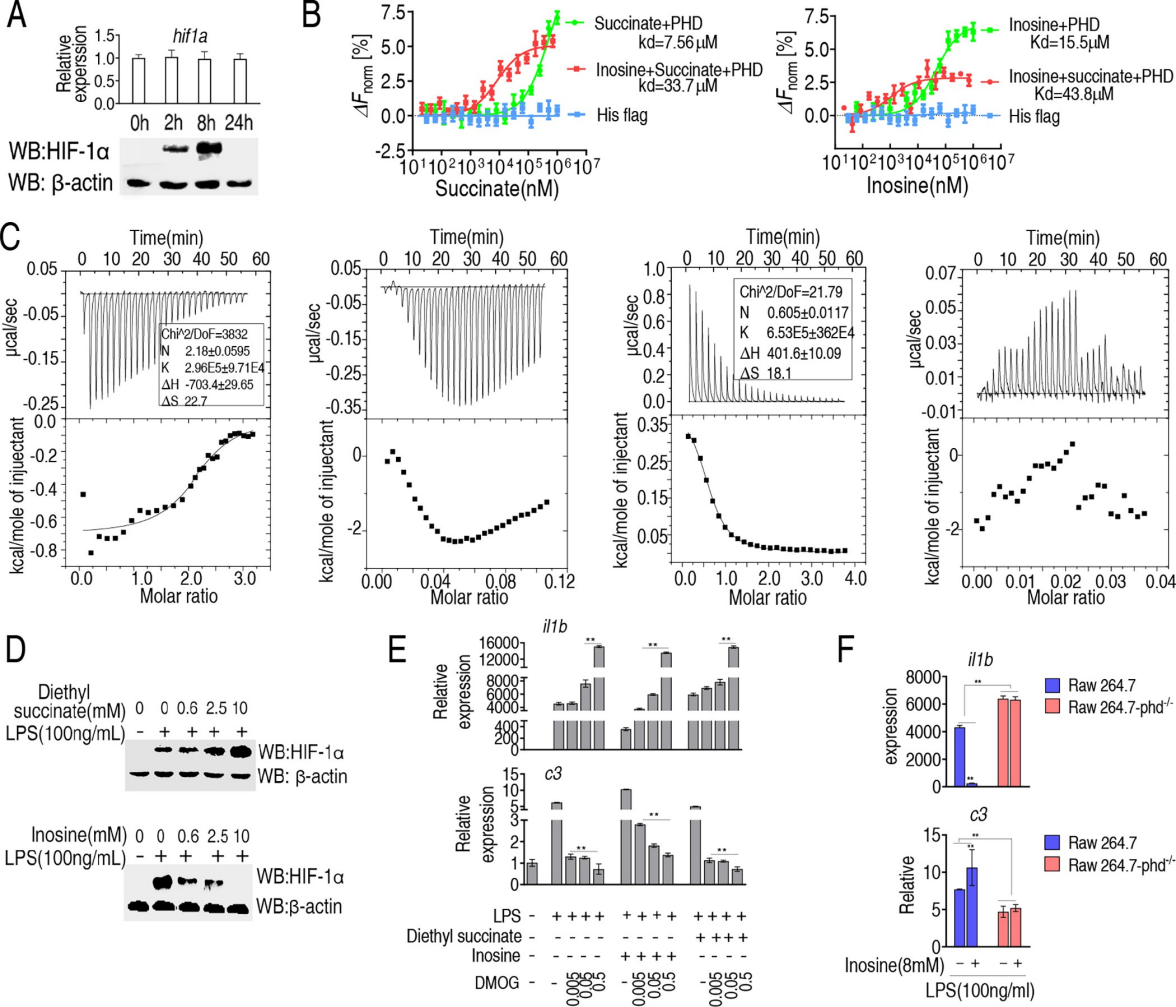
Interactions between PHD, succinate and inosine and impact on HIF 1-α protein abundance. A, qRT-PCR for transcript abundance and Western blot for abundance of HIF 1-α protein at the indicated time points after treatment with LPS. B, MST analysis of PHD plus succinate and/or inosine. C, ITC curves for the binding of succinate (the first panel) or in the presence of inosine (the second panel) or inosine (the third panel) or in the presence of succinate (the forth panel) to PHD. The top panels showed data obtained from automatic injections after baseline corrections. The bottom panels showed the integrated curve that was fitted into a sequential two-site binding model. K: association constant; N: reaction stoichiometry; ΔS: entropy; ΔH: enthalpy. D, Western blot for HIF 1α protein in the presence of succinate or inosine at the indicated doses plus LPS stimulation. E, Transcripts for *il1b* and *c3* in cells exposed to LPS, inosine, succinate and the indicated concentration of DMOG. F, Effect of PHD knock-out on *il1b* and *c3* expression in LPS-treated macrophages in the presence or absence of exogenous inosine. Results (E-F) are displayed as mean ± SEM, and significant differences are identified (*p < 0.05, **p < 0.01) as determined by non-parametric Kruskal-Wallis one-way analysis with Dunn multiple comparison post hoc test for results.

Previous studies indicate that succinate inhibits HIF-1α prolyl hydroxylase (PHD), which destabilizes HIF-1α and promotes its degradation via the proteasome [19, 34]. Here, we proposed and tested the possibility that succinate and inosine antagonistically regulate HIF-1α via PHD. Consistent with this possibility, in LPS-treated RAW264.7-asc cells, the abundance of HIF-1α increased to a peak level and then declined from approximately 8 h after treatment, and correlated positively with expression of IL-1β over the experimental time course, although transcription of HIF-1α was unaffected (Fig 4A). Next, microthermal electrophoresis was used to measure competitive binding and the relative affinities of succinate and inosine for PHD. The results indicate that inosine dramatically decreased the affinity of succinate for PHD (7.65 μM vs. 33.7 μM, respectively) while succinate also decreased the affinity of inosine for PHD (15.5 μM vs. 43.8 μM, respectively) (Fig 4B). Moreover, isothermal titration calorimetry (ITC) was adopted to confirm this conclusion as previously described [35]. During titration, constant volume of metabolites (succinate or inosine or both) were added to PHD protein solutions. The top panels of Fig 4C showed the raw data of heat generation during interaction of PHD with metabolites. The obtained experimental points were then fitted into an integrated curve as shown in the bottom panels of Fig 4C. Thus, PHD exibited a smooth binding isotherm with succinate alone or inosine alone (Fig 4C, the first panel and the third panel, respectively). However, once inosine was present for the titration of PHD with succinate (Fig 4C, the second panel) or succinate was present for the tirtration of PHD with inosine (Fig 4C, the forth panel), the smooth binding isotherm was abroagted, suggesting that inosine and succinate directly competed for binding to PHD.

Consistent with the above observations, succinate appeared to stabilize HIF-1α in LPS-treated RAW264.7-asc cells in a dose-dependent manner, while inosine appeared to destabilize HIF-1α in a dose-dependent manner (Fig 4D). To further confirm this interplay between *il1b* and *c3* expression, RAW264.7-asc cells were treated with PHD inhibitor, dimethyloxallyl glycine (DMOG), in the presence of succinate or inosine. Inosine alone decreased LPS-induced *il1b* expression, which was counteracted by DMOG in a dose-dependent manner. However, succinate-enhanced *il1b* expression was boosted by DMOG in a dose-dependent manner (Fig 4E, upper panel). In contrast, inosine-enhanced *c3* expression was counteracted by DMOG in a time-dependent manner. The presence of both succinate and DMOG reduced *c3* expression similar to non-treated cells (Fig 4E, lower panel). Furthermore, expression of *il1b* was higher and expression of *c3* was lower in LPS-treated PHD-deficient (KO) RAW264.7-asc cells than in LPS-treated PHD-proficient RAW264.7-asc cells independent of the presence of exogenous inosine (Fig 4F). These results support the conclusion that inosine and succinate antagonistically regulate PHD/HIF-1α. We propose this is a highly relevant and important mechanism for coordinating and regulating cellular and humoral phases of the innate immune response to bacterial pathogens.

### Accumulation of ADP and AMP promotes inosine production

**Fig 5:**
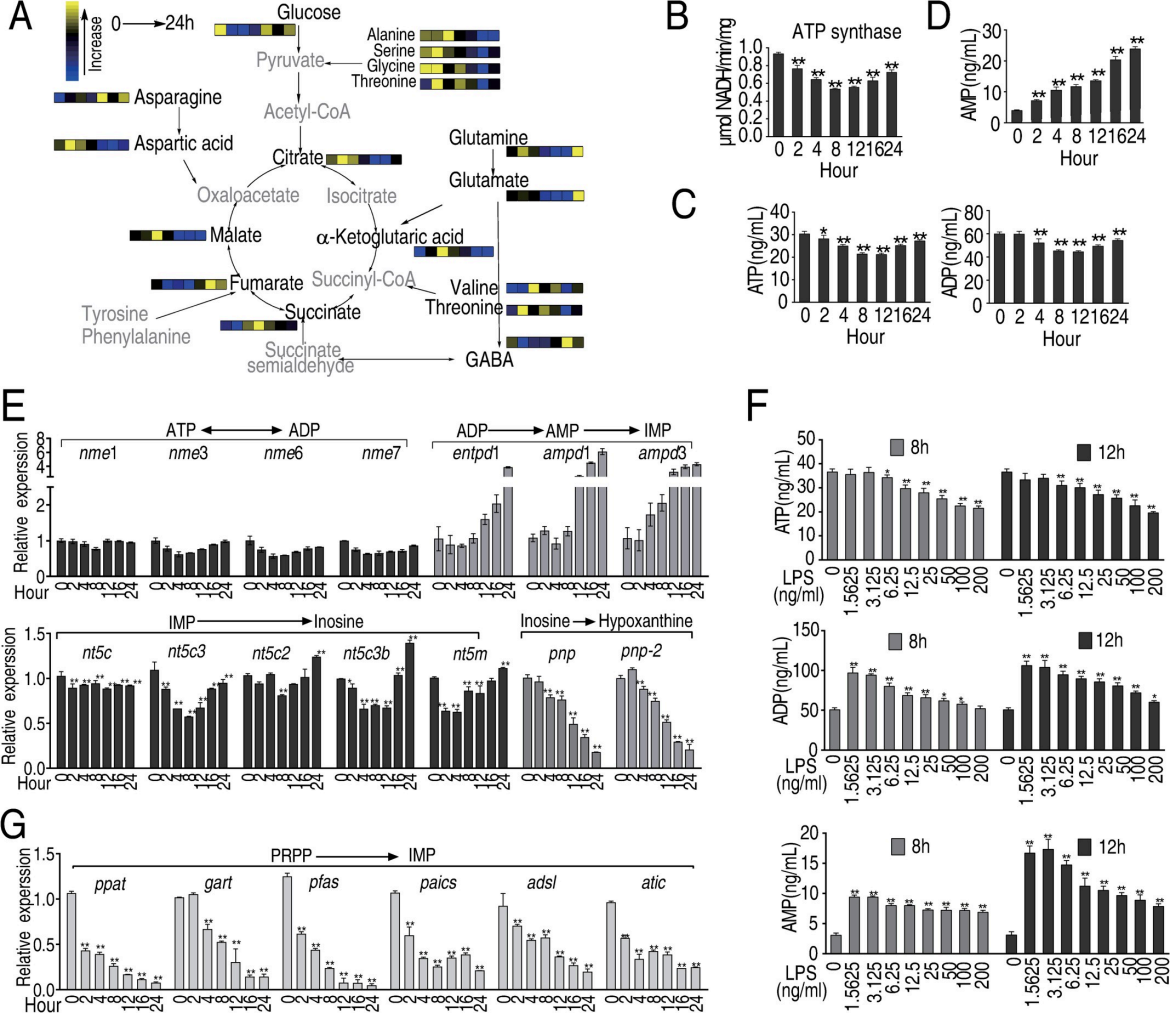
Purine metabolites in LPS-treated macrophages. A, Summary of the abundance of TCA cycle and purine metabolites at the indicated time points after LPS stimulation, based on data (Fig 2A), B, Quantification of ATP synthase activity at the indicated time points in LPS-treated macrophages C, Quantification of ATP and ADP at the indicated time points after LPS stimulation. D, Abundance of AMP at the indicated time points in LPS-treated macrophages. E, Transcription of genes in the ATP salvage pathway in LPS-treated macrophages F, Quantification of ATP, ADP and AMP at 8 and 12 h after exposure to the indicated concentration of LPS. G. Transcripts of genes involved in *de novo* ATP synthesis were quantified by PCR. Results (B-G) are displayed as mean ± SEM, and significant differences are identified (*p < 0.05, **p < 0.01) as determined by non-parametric Kruskal-Wallis one-way analysis with Dunn multiple comparison post hoc test for results.

A previous study reported that the abundance of inosine and other purine metabolites increases in cells treated with LPS [19] and a separate study showed that α-ketoglutarate (α-KG) negatively regulates ATP synthase [36]. Therefore, RAW264.7-asc cells were exposed to LPS, and the abundance of α-KG and other purine metabolites and expression of enzymes involved in purine biosynthetic or salvage pathways were investigated. The results showed that α-KG and succinate respond with similar kinetics to LPS, peaking 4 to 8 h post-treatment (Figs 5A and S7), followed by the lowest activity of ATP synthase at 8 and 12 h (Fig 5B), which may be related to the inhibition of α-KG to ATP synthase (S8 Fig).

In contrast, ATP decreases to a low concentration approximately 8 h post-treatment and then increases from 16 to 24 h post-treatment (Fig 5C). The results showed that α-KG and succinate respond with similar kinetics to LPS, peaking 4 to 8 h post-treatment (Figs 5A and S7), followed by the lowest activity of ATP synthase at 8 and 12 h (Fig 5B), which may be related to the inhibition of α-KG to ATP synthase (S8 Fig). Similar pattern was detected in ATP and ADP kinetics (Fig 5C). However, AMP level increased in a time-dependent manner (Fig 5D). This is consistent with the fact that expression of genes in the inosine salvage pathway as well as inosine respond to LPS with similar kinetics as AMP, while expression of *pnp* and *pnp2*, which converts inosine to hypoxanthine, decreased gradually in cells exposed to LPS (Figs 5E and S9). In addition, expression of genes converting ATP to ADP exhibited the similar pattern as ATP synthase did (Fig 5E and S9). The abundance of ATP followed similar kinetics in response to LPS, while the abundance of ADP and AMP increased with the time (Fig 5F). The abundance of ATP, ADP, and AMP also decreased with increasing concentration of LPS (Fig 5F). *De novo* biosynthesis of inosine from glycine, glutamic acid, aspartic acid, and pyruvate, whose concentration decreased in LPS-treated cells (Fig 2A), also appears to be down-regulated by exposure to LPS with time (Fig 5G), suggesting that *De novo* biosynthesis of inosine is inhibited. In summary, these data suggest that the increasing abundance of inosine could be possibly generated through ADP and AMP biosynthesis and inosine salvage pathway.

### The antagonizing effect with succinate and inosine in mouse and fish models

**Fig 6:**
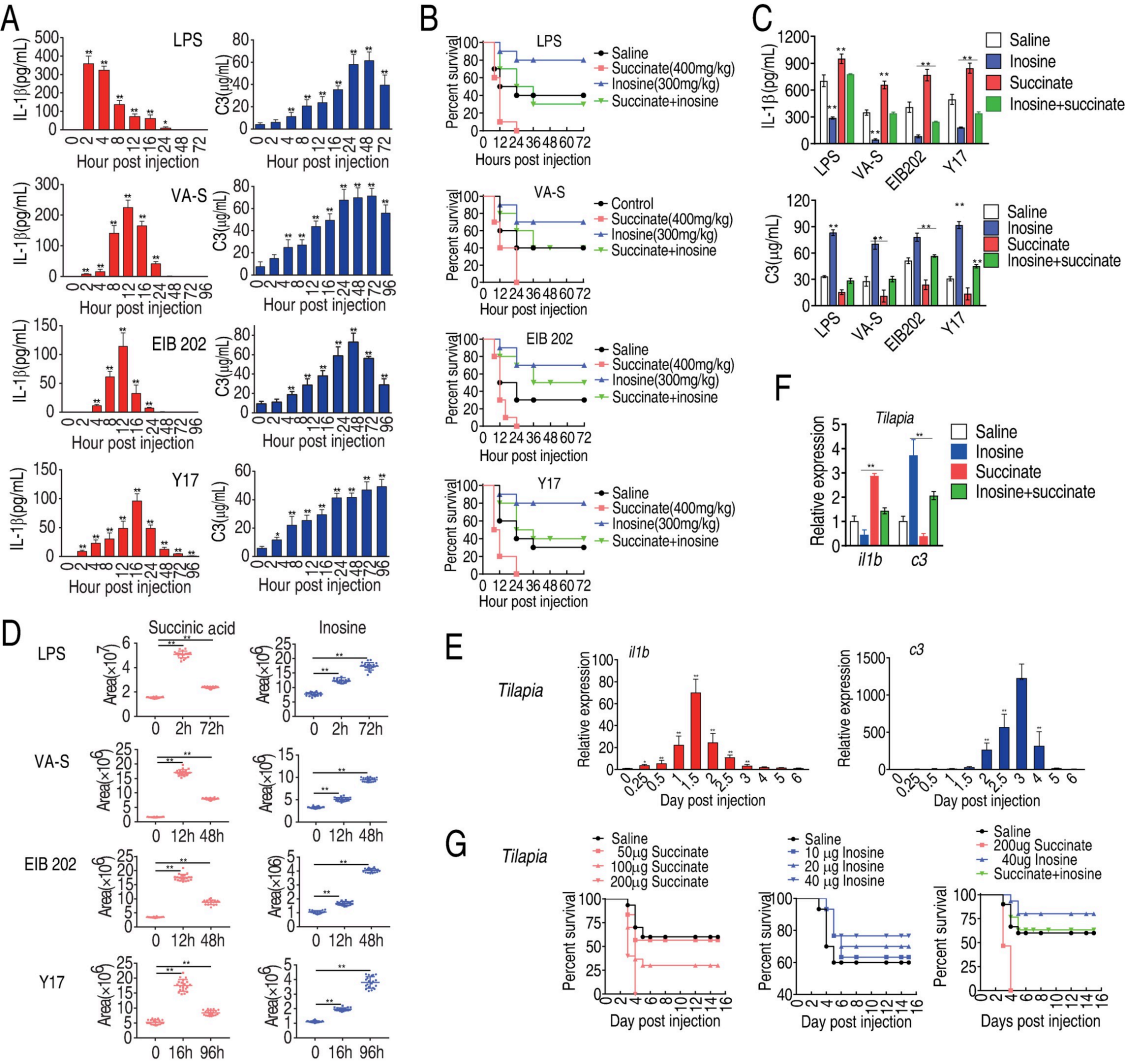
Animal model studies of the impact of succinate and inosine on survival after challenge with bacterial pathogens. A, Quantification of serum level of IL-1β and C3 in mice at the indicated time points after injection with LPS, *V*. *alginolyticus* (VA), *E*. *tarda* EIB202 (EIB202) or *E*. *coli Y17* (Y17), as described in Methods. B, Percent survival of mice co-injected with LPS or the indicated bacterial pathogen and succinate or inosine or both, as indicated. C, Serum abundance of IL-1β and C3 in mice co-injected with LPS or the indicated bacterial pathogen and succinate or inosine or both, as indicated. D, Abundance of inosine or succinate at the indicated time point in mice injected with LPS or the indicated bacterial pathogen. E, Abundance of IL-1β and C3 at the indicated time point after injection of tilapia with *E*. *tarda* (EIB202), as described in Methods. F, Abundance of IL-1β and C3 in tilapia co-injected with EIB202 and saline, succinate or inosine or both, as indicated. G, Percent survival of tilapia co-injected with EIB202 and the indicated amount of succinate or inosine or both. Results (A-F) are displayed as mean ± SEM, and significant differences are identified (*p < 0.05, **p < 0.01) as determined by non-parametric Kruskal-Wallis one-way analysis with Dunn multiple comparison post hoc test for results (A, C-F) and Kaplan-Meier method with log-rank test (B and G).

Finally, the relationships involving IL-1β, C3, succinate, and inosine were investigated in two *in vivo* models, namely: mice infected with *V*. *alginolyticus*, *E*. *tarda* or *E*. *coli*, gram-negative bacterial pathogens associated with sepsis, or the vertebrate tilapia exposed to *E*. *tarda*. First, mice were exposed to LPS (10 mg/kg), extracellular pathogen *V*. *alginolyticus* (1×10^6^ CFU), or intracellular pathogen *E*. *tarda* (1×10^5^ CFU) or *E*. *coli* Y17 (1×10^4^ CFU). Plasma IL-1β and C3 were quantified 0, 2, 4, 8, 12, 16, 24, 48, and 96 h post-infection. After exposure to LPS, IL-1β increased to a peak 2 h post-treatment and then gradually declined to the basal level 48 h post-treatment, while plasma C3 increased to the highest level at 48 h. After exposure to *V*. *alginolyticus* and *E*. *coli*, expression of IL-1β peaked at 12 h and C3 increased until 24 h post-treatment. Mice responded more slowly to *E*. *tarda*, such that IL-1β and C3 peaked at 16 h and 96 h post-treatment, respectively (Fig 6A). This could reflect a different response to extracellular and intracellular pathogens. Taken together, these results are consistent with results in RAW264.7-asc cells presented above, suggesting sequential coordinated activation of cellular (cytokine-mediated) and humoral (complement-mediated) phases of the innate immune response to bacterial pathogens.

To verify the antagonizing effect between inosine and succinate *in vivo*, mice were challenged with the estimated LD_50_ dose of LPS, *V*. *alginolyticus*, *E*. *tarda*, or *E*. *coli*, following by three-days injection of exogenous succinate or inosine or both. Mice were monitored, plasma IL-1β and C3 were quantified and survival was recorded for 72 h post-treatment. All animals died after co-administration of succinate with LPS or the bacterial pathogens. However, 80%, 90%, 70% and 80% survival was detected in co-administration with inosine for mice exposed to LPS, *V*. *alginolyticus*, *E*. *tarda*, or *E*. *coli*, respectively (Fig 6B). When co-administration of both succinate and inosine with LPS or each of the three bacteria, survival rates ranged roughly between those caused by succinic acid and inosine (Fig 6B), showing the antagonizing effect between inosine and succinate. Succinate increased the concentration of IL-1β but decreased C3 in serum for mice exposed to LPS, *V*. *alginolyticus*, *E*. *coli*, and *E*. *tarda*, respectively, while inosine increased expression of C3 and decreased expression of IL-1β in the four treatment groups (Fig 6C). However, inosine antagonized the effect of succinate on IL-1β and C3 expression, where succinate alone decreased C3 expression, and increased IL-1β expression, but these effects were counteracted by inosine (Fig 6C). The abundance of inosine increased from 0 to 48 h post-treatment, while the abundance of succinate increased to an early peak and then decreased at later stages of treatment (Fig 6D), similar to the results obtained in RAW264.7-asc cells (Fig 2).

To extend and confirm these results, a similar experiment was performed in the vertebrate model organism, tilapia, which was challenged by injection with *E*. *tarda* EIB202. After bacterial challenge, expression of *il1b* increased to a peak within 36 h of injection, while *c3* increased gradually until day 3 post-injection and then decreased (Fig 6E). Co-administration of inosine by injection decreased expression of *il1b* and increased expression of *c3*, while co-administration of succinate increased expression of *il1b* and decreased expression of *c3* (Fig 6F). Furthermore, co-administration of succinate decreased tilapia survival in a dose-dependent manner, while co-administration of inosine increased tilapia survival in a dose-dependent manner compared with saline control. Interestingly, co-administration of both succinate and inosine caused tilapia survival to be similar to saline control (Fig 6G). Taken together, the mouse and fish infection models demonstrate that the abuandance of succinate and inosine are strongly associated with the expression of IL-1β, C3 expression and animal mortality, which can be altered by exogenous supplmentation of metabolites. Thus, the interplay between inosine and succinate are critical for animal survival through regulating the expression of IL-1β and C3 expression.

## Discussion

The emergence of multidrug-resistant bacteria and associated difficult-to-treat or untreatable bacterial infections represents a threat to public health worldwide and a challenge to the global research community. In response, there is recent strong interest in and much research activity is focused on exploiting the host cell innate immune response as a novel therapeutic approach [37]. Metabolism has also emerged as a potential tool for modulating the innate immune response to bacterial infection and other types of difficult-to-treat pathologies [37]. Nevertheless, the potential and roles of specific metabolites in modulating innate immune responses remain largely unexplored and poorly understood. In the present study, we identity metabolites that may have potential as therapeutic tools for modulating the innate immune response to bacterial infection.

Results presented here characterize the kinetics of bacterial phagocytosis, in LPS- primed macrophages, and demonstrate that the kinetics of phagocytosis correlates with acute expression of IL-1β, a marker for the cellular cytokine-mediated innate immune response. In contrast, latent expression of C3 increases gradually and follows different, slower kinetics, representing the slightly delayed humoral complement-mediated phase of the innate immune response. This is consistent with the view that acute cellular immunity is induced in the earliest stages of infection, and this process in turn activates the adaptive immune response, as well as the humoral phase of innate immune response, which restricts infection until the adaptive immune response is fully expressed. Results presented here confirm that relative kinetics of the acute cellular and latent humoral phases of the innate immune response are similar in RAW264.7-asc macrophages *in vitro*, and in mice and tilapia *in vivo* (however, the responses are slower in the animal models, as expected). Thus, the cellular component is induced acutely and prior to the humoral component of the innate immune response to bacterial infection.

**Fig 7:**
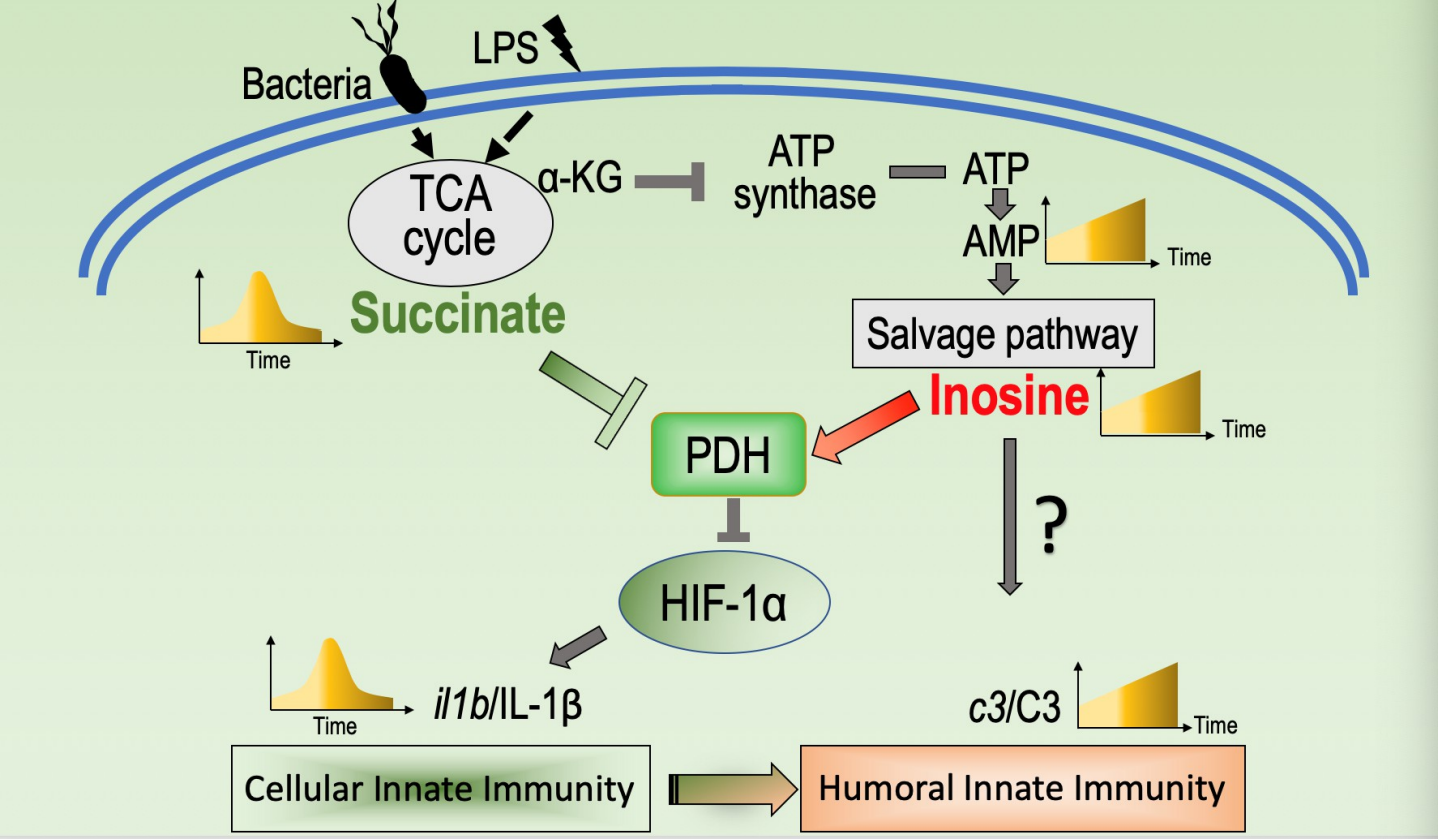
Proposed model. LPS or bacterial infection interrupted the normal TCA cycle that causes the kinetic expression of succinate and inosine, which corresponds the kinetic expression of *il1b*/IL1β and *c3*/C3, respectively, representing a metabolic regulation of the transition from cellular innate immunity to hurmoral immunity. Succinate inhibits the enzymatic activity of PHD that promotes the degradation of HIF-1α at the phase of cellular innate immunity. But inosine competitively bind to PHD to rescue the activity of PHD at the phase of humoral innate immunity to promote the occurrence of humoral innate immunity.

It is well and long ago established that IL-1β and C3 play critical roles in the first line defense against bacterial infection [7,38]. Other studies suggest that IL-1β, TNF-α or IL-6 may also play roles in regulating expression of C3 (and the humoral complement-mediated phase of the innate immune response). However, the precise mechanisms involved and interplay between various cytokine factors in such regulation remains poorly understood [39,40]. Here, to explore a potential role of metabolism in regulating the innate immune response, we characterize the kinetics with which metabolite abundance changes in response to challenge with LPS and/or bacterial pathogens in macrophages and *in vivo* animal systems. The results suggest that succinate and inosine play key roles in modulating cytokine/phagocytosis-mediated cellular innate immunity and humoral complement-mediated innate immunity, respectively. Overactive innate immunity, for example, the high production of IL-1β has been associated with higher sepsis associated mortality [41]. We provide evidence that inosine can down-regulate expression of Il-1β, by a mechanism involving competitive binding of inosine and succinate to PHD, which in turn regulates stability/activity of HIF-1α. In *in vivo* mouse and fish animal model systems, inosine appears to promote humoral complement-mediated innate immunity, attenuate acute cellular innate immunity and increase survival of mice infected with intracellular (EIB202) and extracellular (*E*. *coli* Y17 and *V*. *alginolyticus*) pathogens. Evidence is also presented that inosine accumulates over time in LPS-stimulated macrophages due to increased flux through the inosine salvage pathway. Taken together, the findings presented in this study suggest that the LPS- or bacterial pathogen-induced inflammatory response is modulated and the cellular and humoral phases of the response may be coordinated by dynamic changes in two critical metabolites, succinate and inosine. These novel findings expand our understanding of how metabolism influences immunity and pathogen-induced inflammation. These ideas are summarized in Fig 7.

The core findings of this study are: 1) In response to bacterial challenge, host cells induce an acute cellular macrophage- and cytokine-dependent response followed by a humoral complement-mediated innate immune response. 2) A dynamic metabolome is associated with a dynamic innate immune response, where succinate and inosine appear to play crucial roles in modulating cellular and humoral phases of the response, respectively. These results are consistent with previously presented concept of, a dynamic “anti-infective metabolome” [3]. Thus, our understanding of the interplay between metabolism and immune response is increasing [4,9,14,37], but how the two processes are coordinated remains poorly understood. Nevertheless, the novel roles for inosine and succinate in modulating the innate immune response could be an important step forward. 3) The mechanism by which succinate and inosine antagonistically regulate distinct phases of the innate immune response likely involves competitive binding to PHD, leading to antagonistic regulation of HIF-1α. 4) The immunomodulatory function of inosine was suggested in previous studies of colitis [42] and allergic lung inflammation [43]. However, the present study provides novel evidence that supports possible future clinical use of inosine to modulate the innate immune response, promote proteasome-mediated degradation of HIF-1α, and/or limit adverse consequences of high and/or persistent expression of IL-1β.

Finally, it is worth noting a previous study showing that extracellular inosine exerts its anti-inflammatory activity through the puriorigenic A1/A2 receptor [44]; however, this study did not reveal the mechanism by which inosine modulates the immune response, nor exclude that inosine alters the stability of NF-κB or transcription of upstream kinases mitogen-activated protein kinase, c-Jun N-terminal kinase and c-JUN. In contrast, we found that inosine and succinate competitively regulate PHD. An explanation for this discrepancy awaits further study.

In summary, this study describes novel relationships between a dynamic metabolome and phases of the innate immune response to bacterial infection. Two key metabolites are succinate and inosine, whereby succinate promotes while inosine attenuates expression of IL-1β, via competitive binding and regulation of PHD. Evidence is presented that inosine has the potential to promote survival of mice infected by extracellular and intracellular bacterial pathogens. Therefore, inosine may have potential as a novel therapeutic tool for difficult-to-treat bacterial infections, including bacterial sepsis associated with high levels of IL-1β.

## Materials and methods

### Ethics statement

All the animal work was conducted in strict accordance with the recommendations in the Guide for the Care and Use of Laboratory Animals of the National Institutes of Health. The protocol was approved by the Institutional Animal Care and Use Committee of Sun Yat-sen University (approval no. SYSU-IACUC-2020-B126716).

### Cell culture and quantitative phagocytosis assay

Murine macrophage cell line RAW264.7-asc cell line was a kind gift from Dr. Jiahuai Han, Xiamen University (Xiamen, China) [45]. RAW264.7-asc cells were cultured at 37°C, 5% CO_2_ incubator in DMEM (Hyclone) supplemented with 10% (V/V) cosmic calf (Hyclone), 100 U/ml penicillin G and 100 U/ml streptomycin.

Bacterial phagocytosis by macrophages was examined as described previously [45–47]. Briefly, RAW264.7-asc cells were harvested using CaCl_2_- and MgCl_2_-free PBS containing 5 mM EDTA and plated at 5 × 10^6^ macrophages/well in 6 well plates. For experiments with LPS treatment, the cells were deprived of serum overnight and then alone or additively incubated with LPS (100 ng/mL) (Sigma-Aldrich, St. Louis, MO, USA) for indicated time in serum-starved medium (DMEM/0.5% serum). After LPS treatment, *E*. *coli*-GFP or other FITC-conjugated bacteria were centrifuged onto macrophages at a multiplicity of infection (MOI) of 100 in DMEM without serum. Then the plates were placed at 37°C for 1.5 h. After infection, macrophages were vigorously washed with cold PBS in order to stop additional bacterial uptake or destruction of bacteria in the phagosome. Cells were washed at least four times in cold PBS and subjected to flow cytometry analysis with excitation at 494 nm.

### Bacterial strains and experimental animals

*V*. *alginolyticus* V12G01, *E*. *tarda* EIB202 and *E*. *coli* Y17 were from the bacterial collection of our laboratory. V12G01 were grown in Luria Broth (LB) medium plus additional 3% sodium chloride at 30°C; EIB202 were grown in tryptic soy broth (TSB) at 30°C; Strains of *E*. *coli* were grown in LB at 37°C. All bacteria were grown overnight, diluted 1:100 in 100 mL medium, grown to O.D.600 = 1, washed in sterile saline (0.85% NaCl) and suspended in sterile saline.

Male mice (BALB/c, pathogen-free), weighing 20 ± 2 g from the same litter, were obtained from the Animal Center of Sun Yat-sen University. Mice were reared in cages and fed with sterile water and dry pellets diets.

Juvenile tilapia (body length: 3–4 cm, body weight: 2 ± 0.2 g) were purchased from a commercial breeding corporation (Guangzhou, P.R. China), maintained in 25 L open-circuit water tanks with aeration and determined to be free of *E*. *tarda*. Tilapia were fed on a balanced commercial diet containing 37.53% crude protein, 3.82% crude fat, and 10.79% crude ash related to wet matter, and 6.89% moisture, based on NRC recommendations. Animals were fed with 3% body weight per day.

### Isolation and culture of BMDM

Isolation and culture of BMDM was performed as previously described [24]. Briefly, Balb/c mice were killed by cervical dislocation and soaked in 75% ethanol. Then, femurs and tibias were harvested and the bone marrow cells from all bones were flushed out. After centrifuging for 5 min at 310 × g, erythrocytes were eliminated using Red Blood Cell Lysing Buffer (Sigma-Aldrich, St. Louis, MO, USA). The remaining cells were seeded in plates and incubated in complete medium with 50 mg/mL recombinant mouse M-CSF (R&D Systems, Inc., Minneapolis, MN, USA) for 7 days to form proliferative nonactivated cells.

### RNA extraction and real-time quantitative PCR

Total RNA was extracted from RAW264.7-asc and BMDM cells or fish spleen samples with Trizol (Invitrogen, United States), and the purified RNA was quantified spectrophotometric ally. qRT-PCR was carried out on 1 μg of total RNA by using a Prime Script RT reagent kit with gDNA eraser (TAKARA, Japan) according to manufacturer’s instructions. qRT-PCR was performed in 384-well plates with a total volume of 10 μL containing 5 μL 2× SYBR Premix Ex Taq, 2.6 μL H_2_O, 2 μL cDNA template, and 0.2 μL each of forward and reverse primers (10 μM) (S1 Table). The cycling parameters were listed as follows: 95°C for 30 s to activate the polymerase; 40 cycles of 95°C for 10 s; and 60°C for 30 s. Fluorescence measurements were performed at 72°C for 1 s during each cycle. Cycling was terminated at 95°C with a calefactive velocity of 5°C/s to obtain a melting curve. All qRT-PCR reactions were performed for six biological replicates, and the data were analyzed with Actin, tubulin and GAPDH as reference gene by 2^-ΔΔ^CT method [48].

### GC-MS based metabolomics analysis

GC-MS analysis was carried out with a variation on the two-stage technique as described previously [14,45]. In brief, samples were derivatized and then used to firstly protect carbonyl moieties through methoximation, through a 90 min 37°C reaction with 40 μL of 20 mg/mL methoxyamine hydrochloride (Sigma-Aldrich) in pyridine, followed by derivatization of acidic protons through a 30 min and 37°C reaction with the addition of 80 μL N-methyl-N-trimethylsilyltrifluoroace-tamide (MSTFA, Sigma-Aldrich). The derivatized sample of 1 μL was injected into a 30m × 250 μm i.d. × 0.25 μm DBS-MS column using splitless injection and analysis was carried out by Trace DSQ II (Thermo Scientific). The initial temperature of the GC oven was held at 85°C for 5 min followed by an increase to 330°C at a rate of 15°C min^-1^ then held for 5 min. Helium was used as carrier gas and flow was kept constant at 1 mL min^-1^. The MS was operated in a range of 50–600 m/z.

In data processing, spectral deconvolution and calibration were performed using AMDIS and internal standards. A retention time (RT) correction was performed for all the samples, and then the RT was used as reference against which the remaining spectra were queried and a file containing the abundance information for each metabolite in all the samples was assembled. Metabolites from the GC-MS spectra were identified by searching in National Institute of Standards and Technology (NIST) library used the NIST MS search 2.0. The resulting data matrix was normalized using the concentrations of added internal standards which were subsequently removed so that the data could be used for modeling consisted of extracted compound. The resulting normalized peak intensities form a single matrix with Rt-m/z pairs for each file in the dataset. To reduce between-sample variation we centered the imputed metabolic measures for each tissue sample on its median value and scaled it by its inter-quartile range (IQR) [4,5]. In the integration of proteomic and metabolomics data set, the *z*-score analysis scaled each protein or metabolite according to a reference distribution. The control samples were designated as the reference distribution. Thus, the mean and standard deviation of the control samples was determined for each metabolite or protein. Then each sample was centered by the control mean and scaled by the control standard deviation, per molecule. In this way, we can know at how the molecule expressions deviate from the control state.

### Microscale thermophoresis (MST)

Recombinant PHD protein was labeled in MST-optimized buffer using the Monolith NT Protein Labeling Kit Red-NHS (#MO-L011, Nano temper, Munich, Germany) following the manufacturer’s instructions. PHD (10 μM) was incubated with 30 μM labeling dye for 30 min, followed by 16 serial two-fold dilution into buffer containing inosine or succinate (Sigma) After 5 min incubation, approximately 4 μL of each reaction was enclosed in premium-coated glass capillaries and subject to MST on Monolith NT.115; Nano Temper instrument, at 40% MST power and 40% LED power. Data analysis used MST software (MO. Affinity, Munich, Germany). Three independent samples were analyzed using the signal from Thermophoresis + T-Jump. The data were fitted using graph Pad Prism version 5.

### Isothermal titration calorimetry (ITC)

The thermodynamic parameters for PHD with inosine or succinate were measured by ITC (Nano-ITC,TA Instruments, USA) at 25°C. PHD was dissolved in 10 mM HEPES (pH 7.0) at a concentration of 0.05 mM. Inosine or succinate was dissolved in 10 mM HEPES (pH 7.0) at a concentration of 1 mM. The experiments consisted of a preliminary injection of 5 μL (removed for data treatment), followed by 28 injections of 10 μL with time intervals of 150 s between each injection. All assays were run at 37°C with a stirring speed of 351 rpm. Background subtraction was performed using HEPES titrated with PHD. The raw heat flux-time curves were recorded using Nano Analyze software. Binding isotherms were fitted by nonlinear regression. Stoichiometry of the interaction (n), the equilibrium dissociation constant (K_D_). and the change in enthalpy (△H) were calculated. The Gibbs free energy change (△G) was calculated from △G = -RT InK_D_ and the entropy (△S) from △G = △H-T△S.

### Western blotting

Western blotting was performed as previously described [14]. Briefly, RAW264.7-asc cells were lysed in RIPA lysis buffer and boiled for 10 min. After centrifugation, 25 μg of total protein extract was separated by 12% SDS-PAGE, followed by transferring to PVDF membranes for Western blotting. After blocking with 5% milk dissolved in Tris-buffered saline (TBS) containing 0.05% Tween-20 (TBST) for 1 h at room temperature, the membranes were incubated with indicated with rabbit anti-HIF-1α (Abcam). Then mouse secondary antibody (Xiamen Bosheng Corp.) conjugated with horseradish peroxidase was used to detect the signal. Positive band intensities were detected using a gel documentation system (LAS-3000 Fujifilm Medical Systems, Stamford, CT).

### Quantification of ATP, ADP, AMP content and ATP synthase activity

Cells were collected and lysis by sonic in PBS. Total proteins of 50 μg were applied for ATP, ADP and AMP assay and 100 μg were applied for ATP synthase activity assay. ATP, ADP and AMP were measured by enzyme-linked immunosorbent assay with ELISA kit(Moshake Technology Company, China)and ATP synthase activity was measured by F0F1-ATPase Activity Assay Kit (Nanjing Jiancheng Bioengineering institute, China) accordingly to manufacturer’s instructions. Optical density value was measure at a wavelength of 450 nm for ATP, ADP and AMP or 340 nm for ATP synthase activity in a Microplate reader (Victor X5, Singapore). Concentrations were calculated using a dose-response curve.

### Quantification of IL-1β and C3 in RAW264.7-asc supernatants and mouse serum

IL-1β and C3 concentrations in cell supernatant and serum were measured by enzyme-linked immunosorbent assay with IL-1β ELISA kit (Dakewe Technology Company, China) and C3 ELISA kit (Moshake Technology Company, China), respectively, accordingly to manufacture’s instructions. Optical density value was measure at a wavelength of 450 nm in a Microplate reader (Victor X5, Singapore). Concentrations were calculated using a dose-response curve.

### Generation of PHD knockout cells in RAW264.7-asc cell using CRISPR-Cas9

To generate *phd* (Gene ID: 112405) knockout in RAW-264.7-asc cells, 1 × 10^6^ cells were seeded into a 6-well plate. The PHD sgRNA was designed, synthesized and cloned into the lentiCRISPR v2 plasmid, which was purchased at addgene (Plasmid #52961), at http://crispr.mit.edu/ as previously described [49]. lentiCRISPR v2 was a gift from Feng Zhang (Addgene plasmid # 52961). The plasmid was then transfected into cells via electro transformation. Sequences for sgRNAs used to disrupt the phd gene were as following:

Forward: 5′-CACCGCTCGCGCGTACCGGGCCCGG-3′

Reverse: 5′- AAACCCGGGCCCGGTACGCGCGAGC-3′.

Seventy-two hours after transfection, medium was changed to the same medium containing puromycin (2 μg/mL) and transfected cells were selected for 5 days in the presence of puromycin. Subsequently positive clones were selected through flow cytometry. The presence of indels in each clone was verified by PCR, followed by DNA sequencing. In addition, western blot was used to furtherly confirm the results of PHD knockout cell line.

### Off-target analysis of established cell lines

The potential off-target sites were selected according to online tools at http://crispr.mit.edu/. Six potential off-targets were selected for the sgRNA target site. These sites were verified by PCR, followed by DNA sequencing. The information about the selected off-target sites was listed in (S2 Table).

### Investigation of mouse and fish survival and immune gene expression upon bacterial infection

To investigate effect of exogenous succinate and inosine on survival post bacterial infection and LPS administration, mice were intraperitoneally challenged with LPS (75 mg/kg; n = 20) or *V*. *alginolyticus* V12G01 (3 × 10^8^ cfu/mouse; n = 20) or *E*. *tarda* EIB202 (5 × 10^7^ cfu/mouse; n = 20) or *E*. *coli* Y17 (2 ×10^6^ cfu/mouse; n = 20) or in combination with succinate (400 mg/kg; n = 20) or with inosine (300 mg/kg; n = 20) or both for three days. Survivals were monitored for 72 h because pre-test showed that mouse death mainly happened during 12–48 h and stopped in 72 h when mice were infected the same doses of the pathogen.

Tilapia were divided into 8 groups for the effect of exogenous succinate and inosine on survival post bacterial infection. They were intramuscularly injected with saline or succinate (50 μg, 100 μg or 200 μg; n = 30 for each dose) or inosine (10 μg, 20 μg or 40 μg; n = 30 for each dose) daily for 3 days. Then fish were challenged with *E*. *tarda* (1×10^5^ cfu/fish). Survivals were monitored for a total of 2 weeks. Pre-test showed that fish death mainly happened during 12–24 h and stopped in 72 h when fish were infected the same doses of the pathogen.

To quantify immune gene expression, spleens of fish were collected 0 d, 0.25 d, 0.5 d, 1 d, 1.5 h, 2 d, 2.5 d, 3 d, 4 d, 5 d, and 6 d post-injection and samples were processed for RNA extraction and gene expression by quantitative real-time polymerase chain reaction (qRT-CR).

### Statistical analysis

Data shown are the means ±SEM. Data between 2 groups were analyzed by unpaired t test (Prism 5.0; graph Pad Software, San Diego, CA, USA) if the data were in Gaussian distribution and had equal variance, or by unpaired t test with Welch’s correction (Prism 5.0; graph Pad Software) if the data were in Gaussian distribution but with unequal variance, or by nonparametric test (Mann-Whitney U test, Prism 6.0; graph Pad Software) if the data were not normally distributed. Data among more than 2 groups were analyzed by the one-way ANOVA followed by Dennett multiple comparisons (Prism 5.0; graph Pad Software) if the data were in Gaussian distribution and had equal variance or analyzed by nonparametric Kruskal-Wallis one-way analysis with Dunn multiple comparison post hoc test (Prism 5.0; graph Pad Software) if the data were not normally distributed. The Gaussian distribution of data was analyzed by D’Agostino‐Pearson omnibus normality test (Prism 5.0; graph Pad Software) and Kolmogorov‐Smirnov test (Prism 5.0; graph Pad Software). The variance of data was analyzed by homogeneity of variance test (SPSS 22.0) or Brown‐Forsythe test (Prism 6.0; graph Pad Software). Statistical details of all experiments can be found in the figure legends and significance is described in the figure legends as: * p < 0.05, ** p < 0.01.

## Supporting information

S1 Fig (Related to Fig 2). **Metabolic profiling of RAW264.7 cells after LPS treatment.** A, Reliability of technical repeats. b, Functional categories of different abundance of metabolites.
(TIF)

S2 Fig (Related to Fig 2). **Metabolic profiling of RAW264.7 cells treated with LPS at different time points as indicated**. Z-score plots corresponding to Fig 2A of significantly differential metabolites (Wilcoxon P<0.01) of 2h, 4h, 8h, 12h, 16h and 24h at the top of the figure indicated, compared with 0h. Metabolites are showed on the y axis.
(TIF)

S3 Fig (Related to Fig 2). Pathway analysis of differential abundance. a, Enriched pathway of metabolites of differential abundance. b, The abundance of differential metabolites in the enriched pathways in (a).
(TIF)

S4 Fig (Related to Fig 2). Scatter plot showing normalized abundance of aspartic acid, myo-inositol, oxalic acid, glycine, adenosine, octadecanoic acid, threonine, desmosterol, hexadecenoic acid, itaconic acid, palmitic acid and monolinolein at different time points (0h, 2h, 4h, 8h, 12h, 16h, and 24h) post LPS treatments.
(TIF)

S5 Fig (Related to Fig 2). **Succinate, inosine, *il1b*, and *c3* kinetics of BMDM cells treated with LPS** Results are displayed as mean ± SEM, and significant differences are identified (*p < 0.05, **p < 0.01) as determined by non-parametric Kruskal-Wallis one-way analysis with Dunn multiple comparison post hoc test for results.
(TIF)

S6 Fig (Related to Fig 3). **qRT-PCR for expression of *il1b* (A) and *c3* (B) in th presence of LPS, inosine or succinate.** Results are displayed as mean ± SEM, and significant differences are identified (*p < 0.05, **p < 0.01) as determined by non-parametric Kruskal-Wallis one-way analysis with Dunn multiple comparison post hoc test for results.
(TIF)

S7 Fig (Related to Fig 5). Relative abundance of in RAW264.7-asc cells exposed to LPS at the indicated concentration. ATP synthase activity in the presence of a-KG.
(TIF)

S8 Fig (Related to Fig 5). **Measurement of intracellular ATP synthase activity using cell lysis plus the indicated concentration of α-Ketoglutarate (n = 3).** Results are displayed as mean ± SEM, and significant differences are identified (*p < 0.05, **p < 0.01) as determined by non-parametric Kruskal-Wallis one-way analysis with Dunn multiple comparison post hoc test.
(TIF)

S9 Fig (Related to Fig 5). Transcription of part genes in the ATP–AMP salvage pathway in LPS-treated macrophages.
(TIF)

S1 Table The primers in this study. (DOCX)

S2 Table Selected Off-target sites. (DOCX)
