# Supplementary figures and images for: Succinate and inosine coordinate innate immune response to bacterial infection
Source: PLoS Pathog. 2022 Aug 26;18(8):e1010796. doi: 10.1371/journal.ppat.1010796 (PMC9455851; doi:10.1371/journal.ppat.1010796)

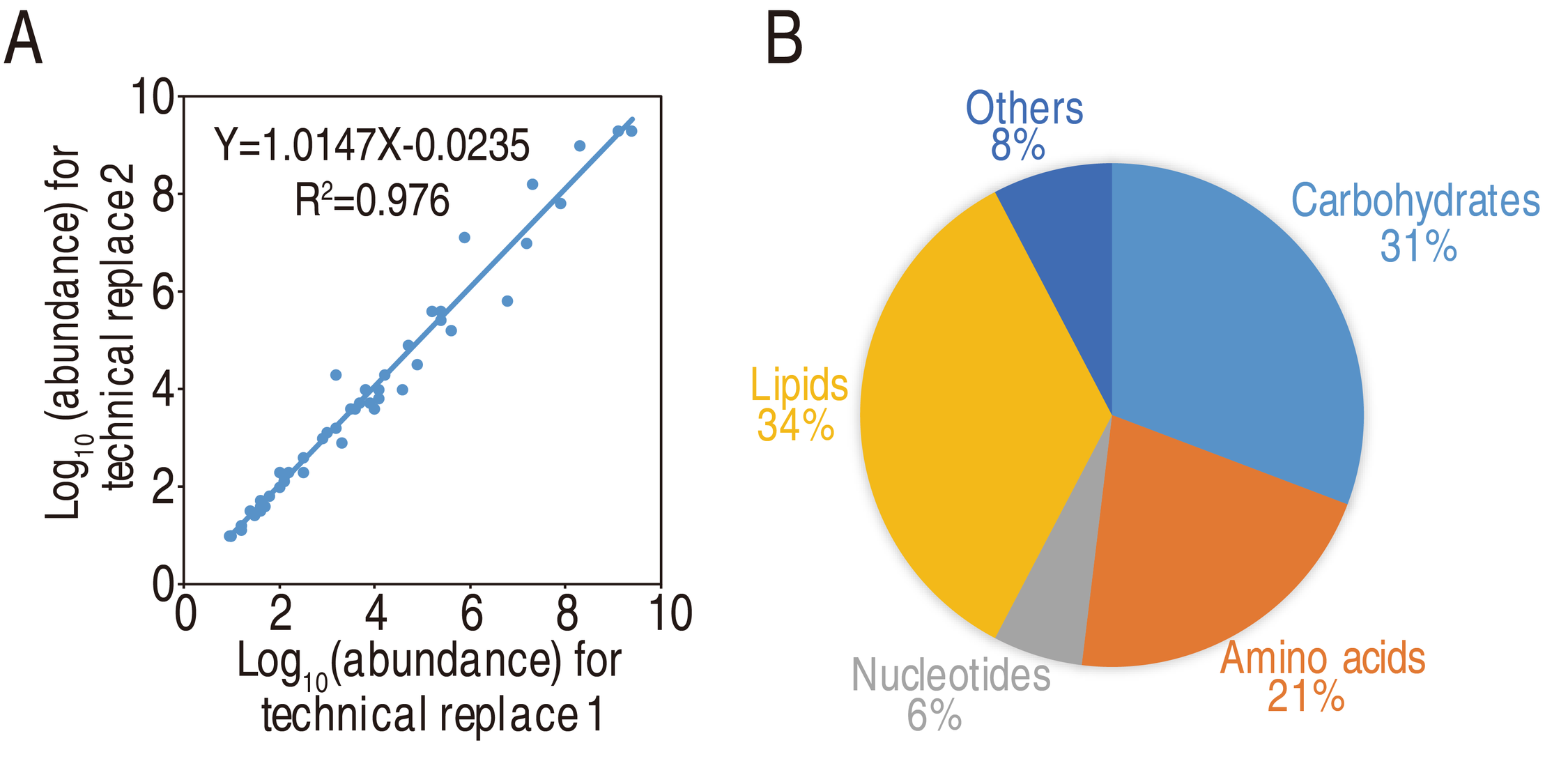

Supplement: S1 Fig — Metabolic profiling of RAW264.7 cells after LPS treatment. A, Reliability of technical repeats. b, Functional categories of different abundance of metabolites. (TIF) [file ppat.1010796.s001.tif]

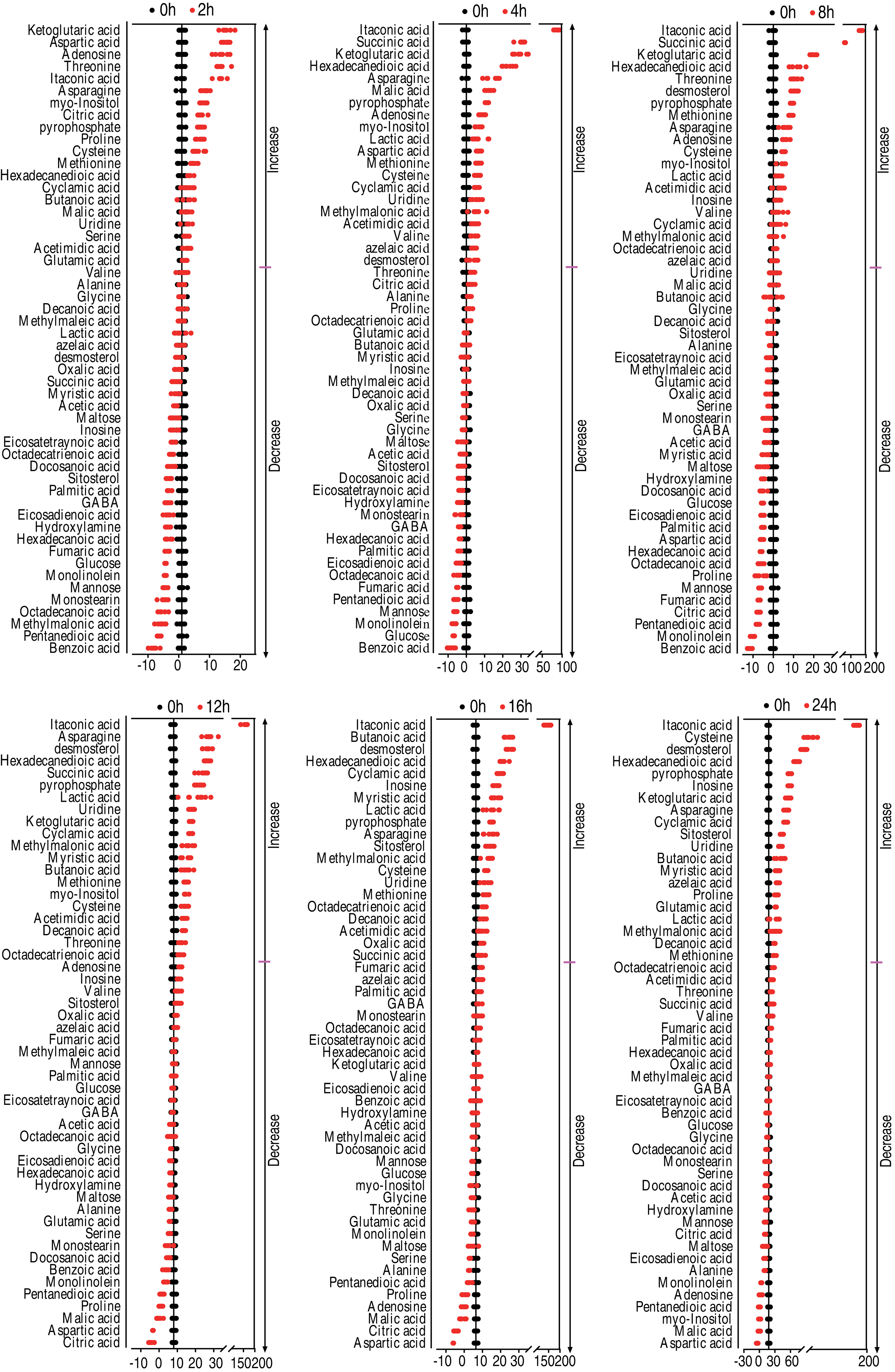

Supplement: S2 Fig — Metabolic profiling of RAW264.7 cells treated with LPS at different time points as indicated. Z-score plots corresponding to Fig 2A of significantly differential metabolites (Wilcoxon P<0.01) of 2h, 4h, 8h, 12h, 16h and 24h at the top of the figure indicated, compared with 0h. Metabolites are showed on the y axis. (TIF) [file ppat.1010796.s002.tif]

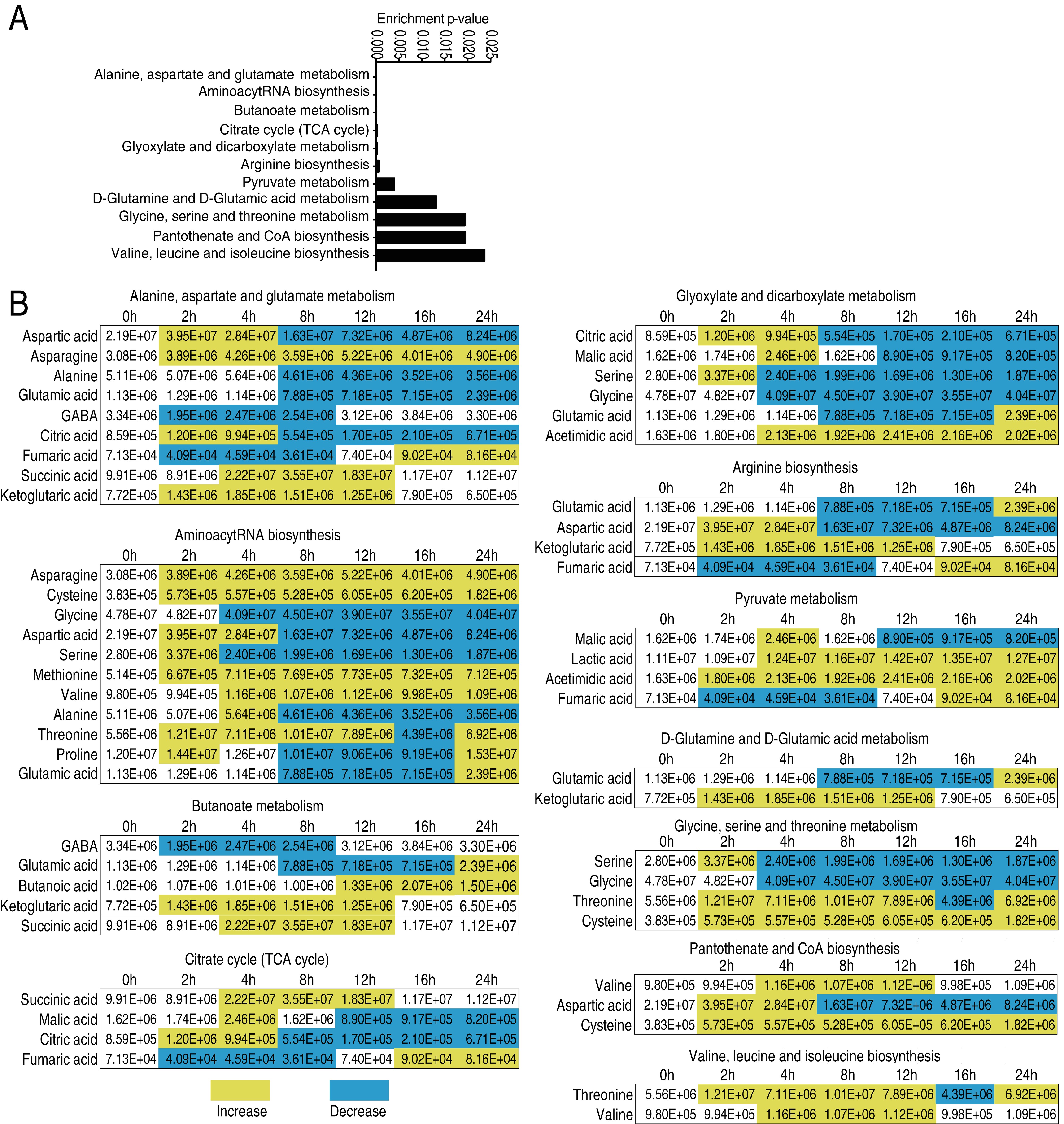

Supplement: S3 Fig — Pathway analysis of differential abundance. a, Enriched pathway of metabolites of differential abundance. b, The abundance of differential metabolites in the enriched pathways in (a). (TIF) [file ppat.1010796.s003.tif]

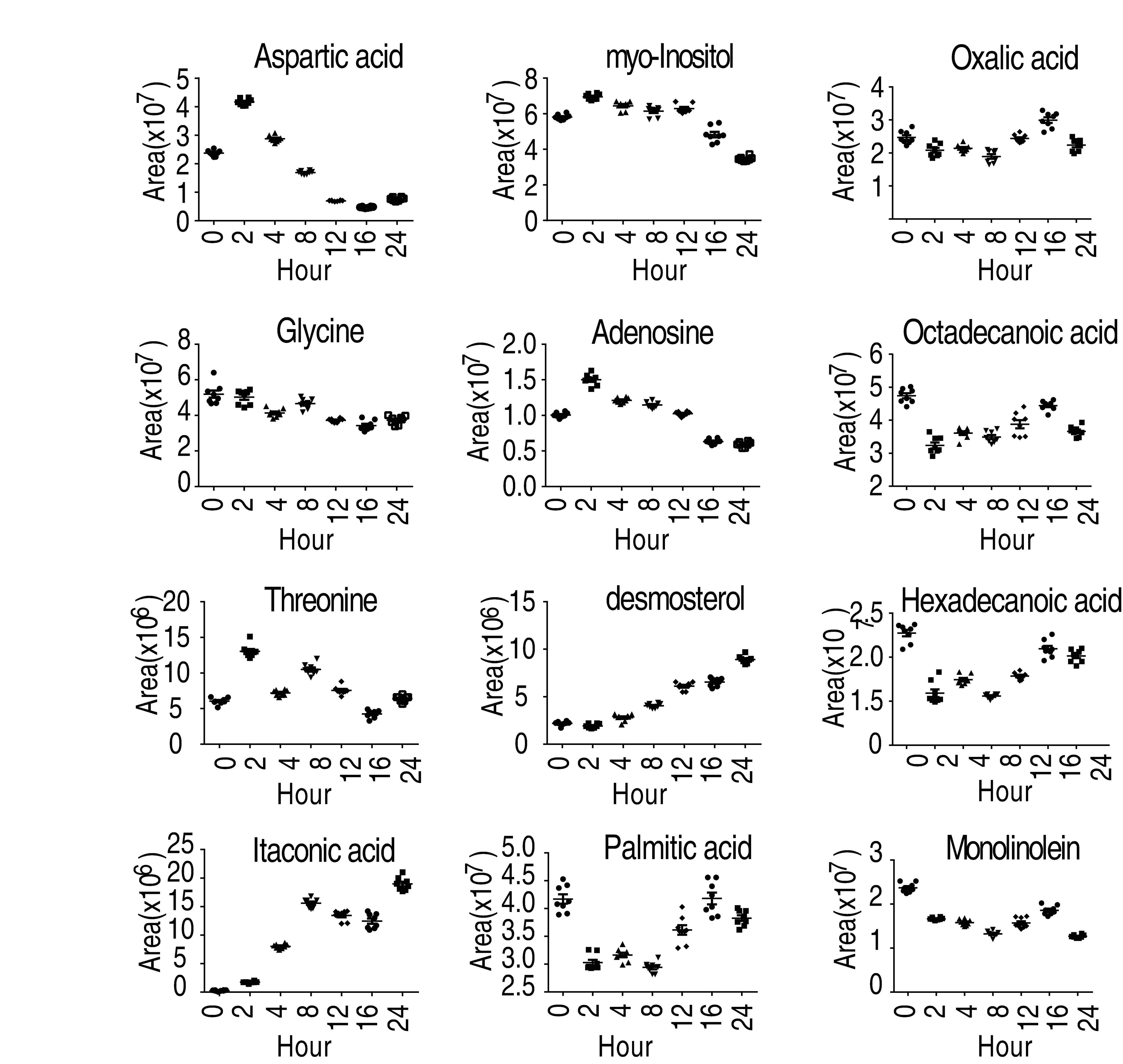

Supplement: S4 Fig — Scatter plot showing normalized abundance of aspartic acid, myo-inositol, oxalic acid, glycine, adenosine, octadecanoic acid, threonine, desmosterol, hexadecenoic acid, itaconic acid, palmitic acid and monolinolein at different time points (0h, 2h, 4h, 8h, 12h, 16h, and 24h) post LPS treatments. (TIF) [file ppat.1010796.s004.tif]

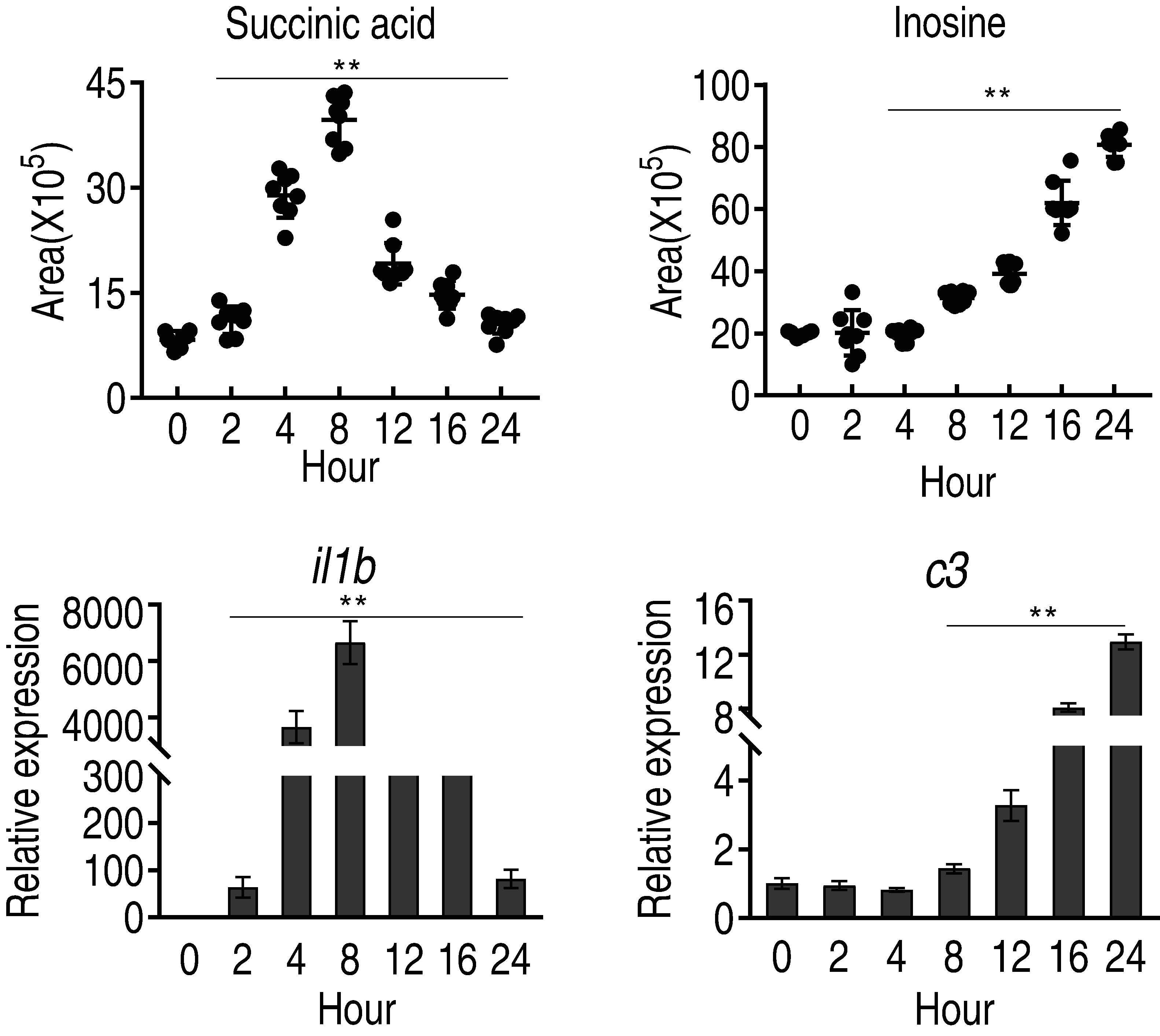

Supplement: S5 Fig — Succinate, inosine, il1b, and c3 kinetics of BMDM cells treated with LPS Results are displayed as mean ± SEM, and significant differences are identified (*p < 0.05, **p < 0.01) as determined by non-parametric Kruskal-Wallis one-way analysis with Dunn multiple comparison post hoc test for results. (TIF) [file ppat.1010796.s005.tif]

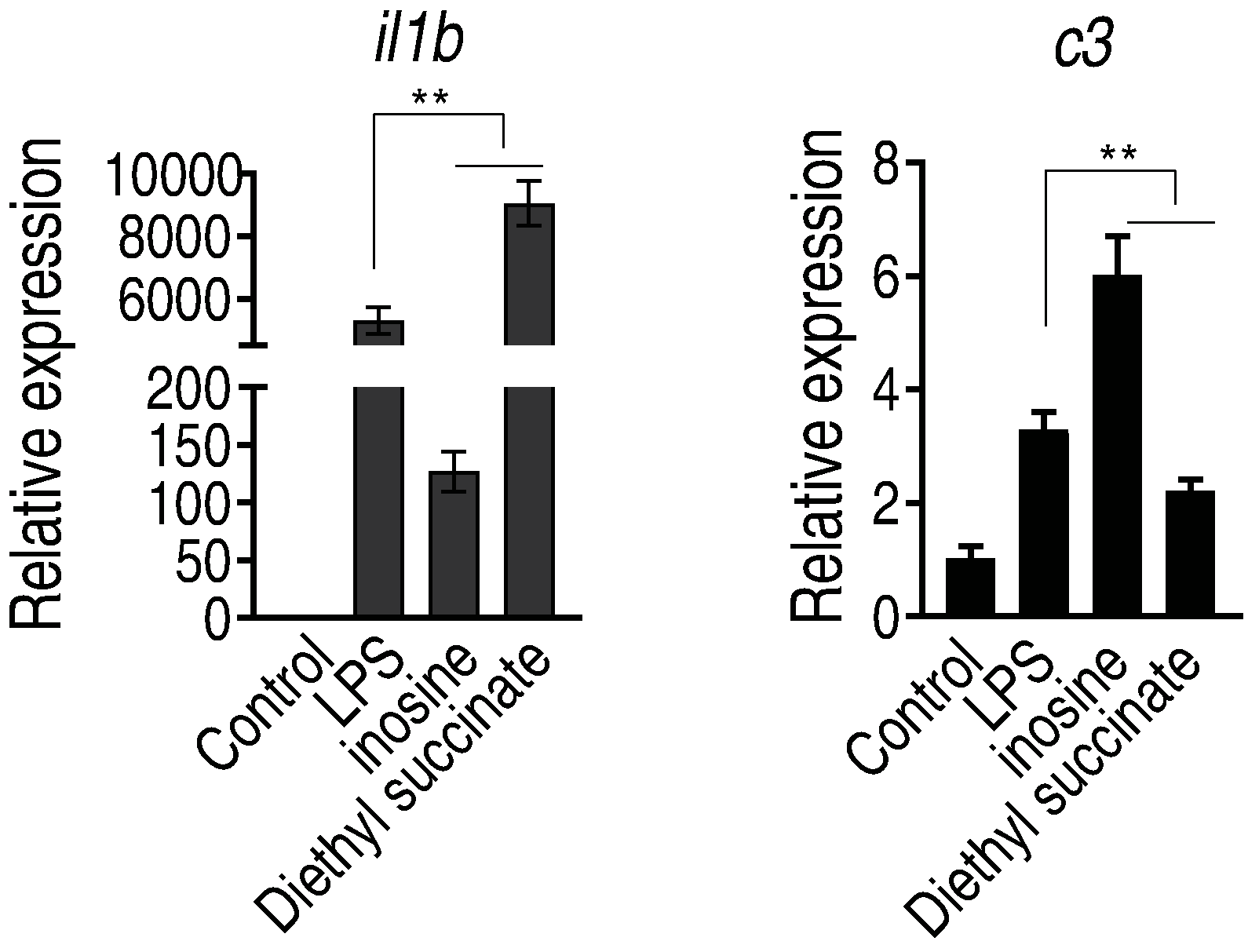

Supplement: S6 Fig — qRT-PCR for expression of il1b (A) and c3 (B) in th presence of LPS, inosine or succinate. Results are displayed as mean ± SEM, and significant differences are identified (*p < 0.05, **p < 0.01) as determined by non-parametric Kruskal-Wallis one-way analysis with Dunn multiple comparison post hoc test for results. (TIF) [file ppat.1010796.s006.tif]

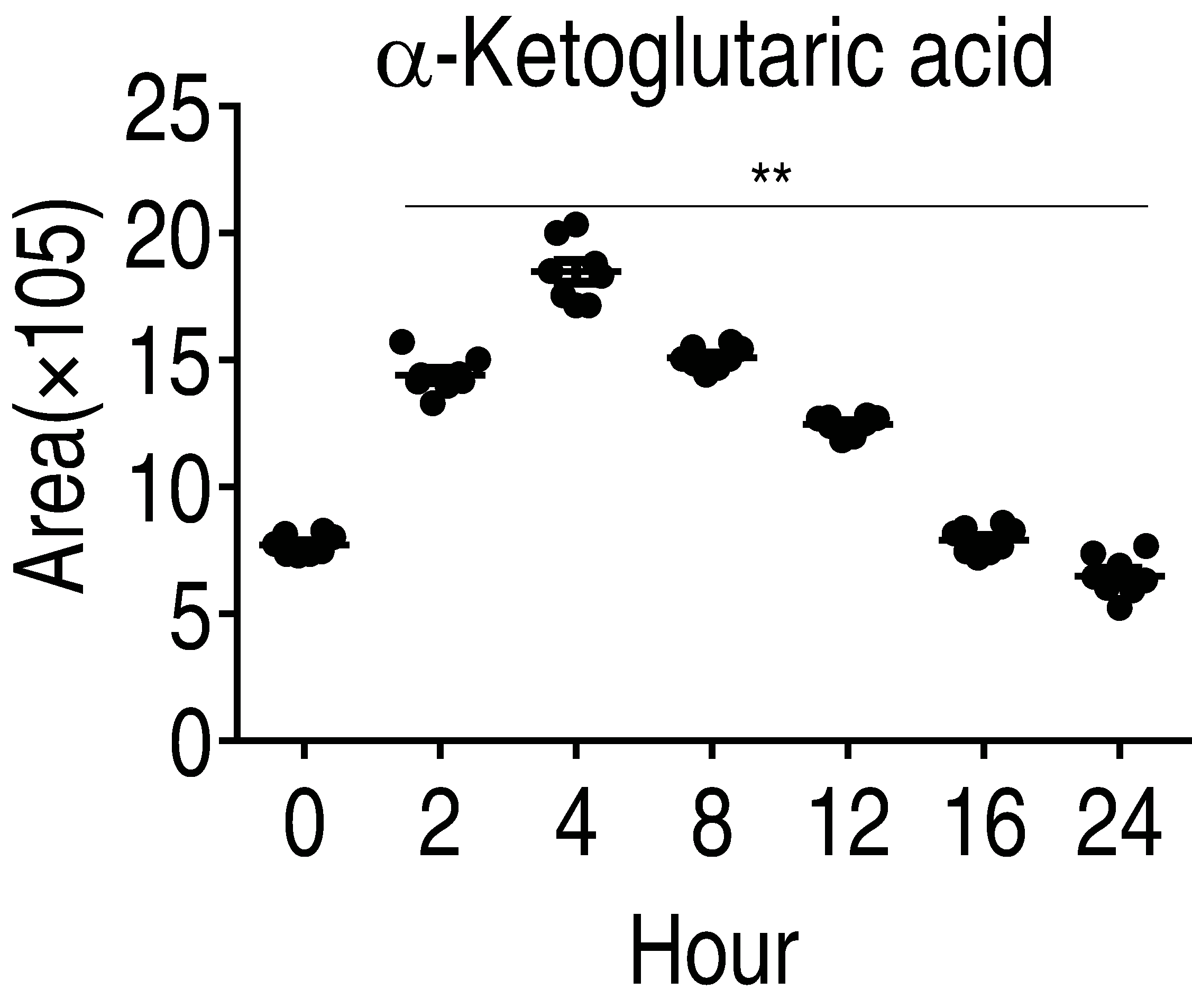

Supplement: S7 Fig — Relative abundance of in RAW264.7-asc cells exposed to LPS at the indicated concentration. ATP synthase activity in the presence of a-KG. (TIF) [file ppat.1010796.s007.tif]

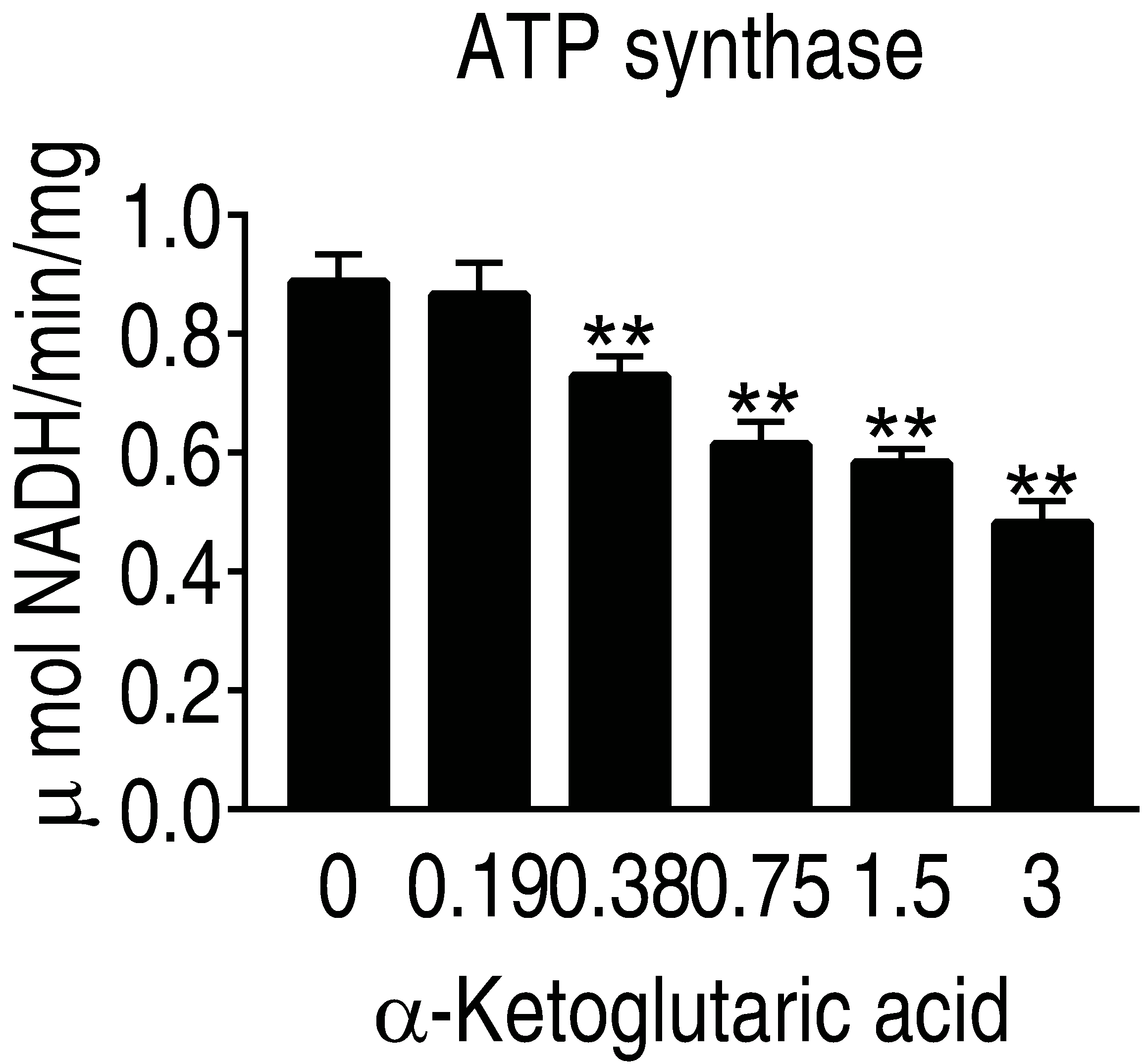

Supplement: S8 Fig — Measurement of intracellular ATP synthase activity using cell lysis plus the indicated concentration of α-Ketoglutarate (n = 3). Results are displayed as mean ± SEM, and significant differences are identified (*p < 0.05, **p < 0.01) as determined by non-parametric Kruskal-Wallis one-way analysis with Dunn multiple comparison post hoc test. (TIF) [file ppat.1010796.s008.tif]

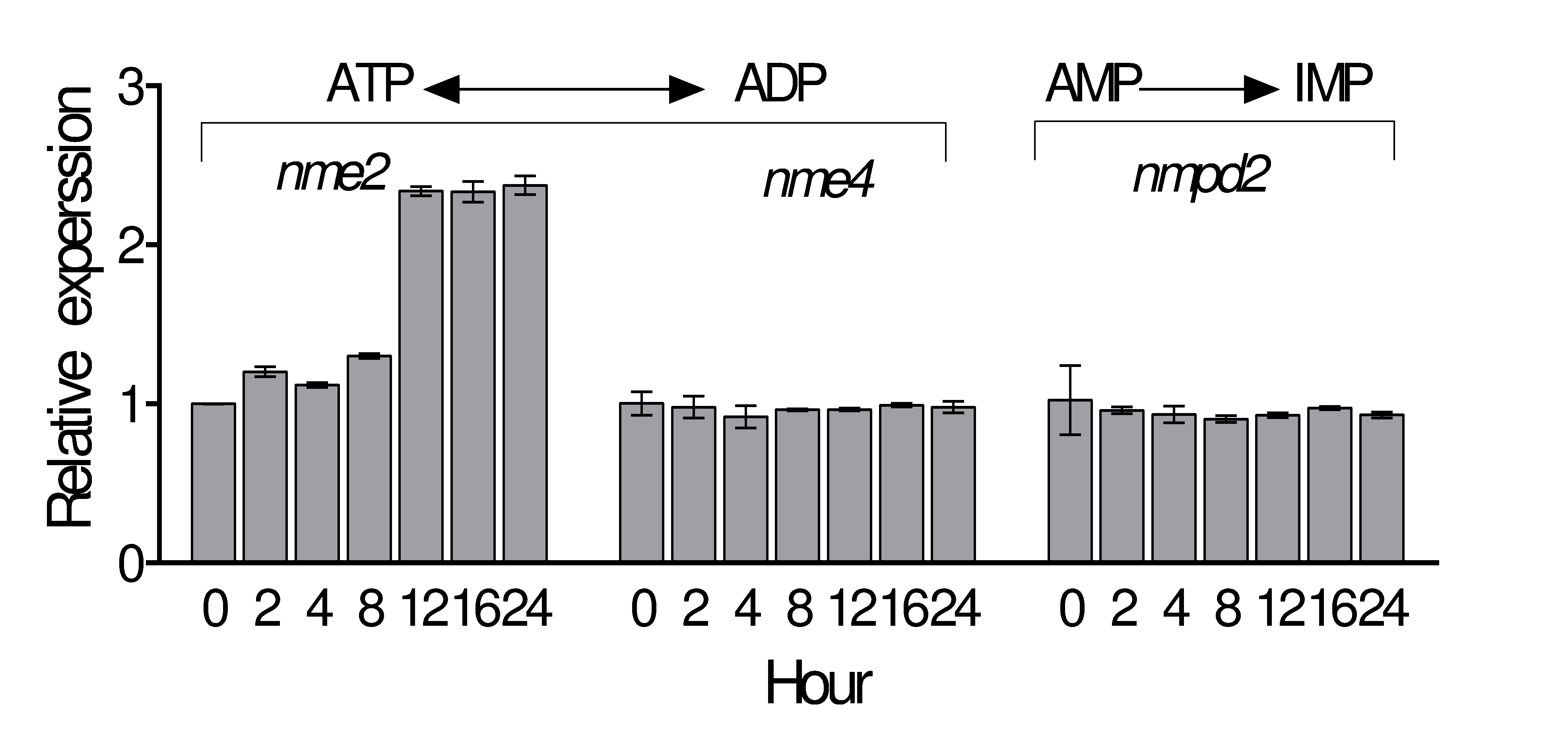

Supplement: S9 Fig — Transcription of part genes in the ATP–AMP salvage pathway in LPS-treated macrophages. (TIF) [file ppat.1010796.s009.tif]
