# Supplementary material for: Succinate and inosine coordinate innate immune response to bacterial infection
Source: PLoS Pathog. 2022 Aug 26;18(8):e1010796. doi: 10.1371/journal.ppat.1010796 (PMC9455851; doi:10.1371/journal.ppat.1010796)
Supplement: S1 Table — (DOCX) [file ppat.1010796.s010.docx]

**S1 Table The primers in this study**

| Species | Gene | Primer | Primer sequence |
| --- | --- | --- | --- |
| mouse | *Ppat* | Forward | tgggtagcaagaattaagaact |
|  |  | Reverse | cccatccttcagtttctgag |
| mouse | *Gart* | Forward | ccaatgctgctgtctctgtc |
|  |  | Reverse | ggcggtccataaactctttagc |
| mouse | *Pfas* | Forward | cactggagacaagagaattgtact |
|  |  | Reverse | cctgtcgcacactcagttct |
| mouse | *Paics* | Forward | ctcattgctgcaaaattctg |
|  |  | Reverse | tcaacaccaaattcaatcttca |
| mouse | *Adsl* | Forward | agacaatacggacctgattattc |
|  |  | Reverse | caggctgaaagtgtgtgaaac |
| mouse | *Atic* | Forward | gcctggcagtcagagatgt |
|  |  | Reverse | ctgcctcaacagttacatctg |
| mouse | *Ampd1* | Forward | cacaagggcctgttaagact |
|  |  | Reverse | ctgcatggatgtgagtatcc |
| mouse | *Ampd2* | Forward | gagatcgctgaggagttgtt |
|  |  | Reverse | ggctcgaagaagaatatctg |
| mouse | *Ampd3* | Forward | tacgtgactgggtttgacag |
|  |  | Reverse | gaaggcagacactaggtgag |
| mouse | *Pkm* | Forward | aagaagggagccactctgaag |
|  |  | Reverse | gtcagcgcctttctccttc |
| mouse | *Pklr* | Forward | aggcgtgaagaagtttgatg |
|  |  | Reverse | ccagacagcatgatacagtcag |
| mouse | *Nme1* | Forward | agggtctgaatgtggtgaag |
|  |  | Reverse | tctgcgctctttacagaatc |
| mouse | *Nme2* | Forward | agaacacctgaagcagcattac |
|  |  | Reverse | cactgccatgaatgatgttc |
| mouse | *Nme3* | Forward | ccgactggttaaatacatgag |
|  |  | Reverse | aagagcgatttctctgtgag |
| mouse | *Nme4* | Forward | ccccaatgtggtccatatct |
|  |  | Reverse | tctgcccagttcaacagttc |
| mouse | *Nme6* | Forward | ggaggctgttcatcagcag |
|  |  | Reverse | gcccacttgtcatgaactc |
| mouse | *Nme7* | Forward | atatggcacccaagaatcag |
|  |  | Reverse | gctgtcgagaaaacacattg |
| mouse | *Ak9* | Forward | ggagctatgctgcagtcact |
|  |  | Reverse | ttggcataaatcgtagtcactg |
| mouse | *Ada* | Forward | ctatgagggcgcagtaaag |
|  |  | Reverse | aggggcagacctcaaagtg |
| mouse | *Dck* | Forward | tgactgcatgaatgaaacag |
|  |  | Reverse | aaagctggttttcagagtcc |
| mouse | *Adk* | Forward | aagcgctgagtgaaaatgtg |
|  |  | Reverse | ccactgagccactttcattg |
| mouse | *Entpd1* | Forward | tcttgggaagagacaaagac |
|  |  | Reverse | atcttgcccataaaatgaat |
| mouse | *Entpd3* | Forward | aggtggcttctgtgtttgac |
|  |  | Reverse | ctcgctccaagtgtgtctg |
| mouse | *Entpd8* | Forward | accaagtttgggattgtgtt |
|  |  | Reverse | gcctgagagctgttcttctg |
| mouse | *Entpd2* | Forward | gatgtgcccaaagacagat |
|  |  | Reverse | ccagccatacttgatgaagt |
| mouse | *Enpp1* | Forward | tcgctggttttgtcagtatg |
|  |  | Reverse | ccgcacctgaatttgttg |
| mouse | *Enpp3* | Forward | accagtcagtgctggagtaatt |
|  |  | Reverse | gtcatgtattccactctatcacag |
| mouse | *Ntpcr* | Forward | tggccccaaacacagagt |
|  |  | Reverse | tccctggtgacattgaacac |
| mouse | *Hprt* | Forward | attaaagcactgaatagaaatagtg |
|  |  | Reverse | ctccaccaataacttttatgtc |
| mouse | *Pnp* | Forward | tgctgatccgtgatcacat |
|  |  | Reverse | ctctgccacagtctcaaagt |
| mouse | *Pnp2* | Forward | gcaggtcccaactttgagac |
|  |  | Reverse | ggccttctccaagttctcat |
| mouse | *Nt5m* | Forward | gggagtcaaaggatttcttc |
|  |  | Reverse | cccaggcatacttctcatag |
| mouse | *Nt5c3* | Forward | ctgccaaacttcagataataac |
|  |  | Reverse | ccatgtaagggaacttctcttc |
| mouse | *Nt5c1a* | Forward | gggagttcgtctcatcaacagt |
|  |  | Reverse | ctgcatcggctgacaagtagag |
| mouse | *Nt5e* | Forward | tcaaatccagggacaaatttag |
|  |  | Reverse | tcatctgcggtgactatgaatg |
| mouse | *Nt5c* | Forward | gtggagcggattatcttgac |
|  |  | Reverse | ccccctccagttgtcactc |
| mouse | *Nt5c3b* | Forward | tgcccttcttcccacaatat |
|  |  | Reverse | gaggcagcttctctttgatg |
| mouse | *Nt5c1b* | Forward | cgtcaatcactatggtctactgat |
|  |  | Reverse | atccttggcaatatgttcag |
| mouse | *Nt5c2* | Forward | ctatcaccgggtgtttgtg |
|  |  | Reverse | tggtagggaatgtagaatcatatg |
| mouse | *il1b* | Forward | tcaggcaggcagtatcactc |
|  |  | Reverse | gaggatgggctcttcttcaa |
| mouse | *atnb* | Forward | ctgtggcatccatgaaactac |
|  |  | Reverse | gaggagcaatgatcttgatct |
| mouse | *c3* | Forward | catcctgcactcaggtagtg |
|  |  | Reverse | gcattagatccctgagtgac |
| tilapia | *ACTB* | Forvard | aatcgtgcgtgacatcaaag |
|  |  | Reverse | gacgtcgcacttcatgatg |
| tilapia | *Gapdh* | Forvard | gccaaggtcataaacgaca |
|  |  | Reverse | aggcagggatgatgttctg |
| tilapia | *il1b* | Forvard | aaggcacaaacctctatctg |
|  |  | Reverse | tgtcgcgtttgtagaagaga |
