# Supplementary material for: Succinate and inosine coordinate innate immune response to bacterial infection
Source: PLoS Pathog. 2022 Aug 26;18(8):e1010796. doi: 10.1371/journal.ppat.1010796 (PMC9455851; doi:10.1371/journal.ppat.1010796)
Supplement: S2 Table — (DOCX) [file ppat.1010796.s011.docx]

**S2 Table. Selected Off-target sites**

| Name | Locus | Sequence | Mismatch | Indel |
| --- | --- | --- | --- | --- |
| *phd* sgRNA2 Target |  | CACAAGCTGGTGTGCCAGGG |  |  |
| *phd* sgRNA2 Off target 1 | chr3:-1145490502 | CAGCAGCTGGTCTGCCAGGG | 3 | ND |
| *phd* sgRNA2 Off target 2 | chr9:166090825 | CCCCAGCAGCTGTGCCAGGG | 4 | ND |
| *phd* sgRNA2 Off target 3 | chr7:-138145062 | CCAGGGCTGGTGTGCCAGGG | 4 | ND |
| *phd* sgRNA2 Off target 4 | chr17:-124629790 | CCTTTGCTGGTGTGCCAGGG | 4 | ND |
| *phd* sgRNA2 Off target 5 | chr15:198011950 | CACAGGCTGCTGTGCCCGGG | 3 | ND |
| *phd* sgRNA2 Off target 6 | chr9:-177791059 | CACAAGCTGGAGGGCCAGGG | 2 | ND |
